# Cholesterol surface-modified oncolytic adenovirus enriched with apolipoprotein E penetrates the blood-brain barrier to target glioblastoma immunotherapy

**DOI:** 10.1016/j.mtbio.2025.102319

**Published:** 2025-09-18

**Authors:** Aodi Niu, Yuqing Lv, Yuxuan Chen, Yuxuan Liu, Chengjian Luo, Mengqing Zheng, Yeyu Shen, Junjia He, Dongni Yao, Huanrong Lan, Hai Zou, Tong Ge, Xiaozhou Mou

**Affiliations:** a School of Pharmacy, Hangzhou Normal University, Hangzhou, 311121, China; b Center for Rehabilitation Medicine, Rehabilitation and Sports Medicine Research Institute of Zhejiang Province, Department of Rehabilitation Medicine, Clinical Research Institute, Zhejiang Provincial People’s Hospital, Affiliated People’s Hospital, Hangzhou Medical College, Hangzhou, 310014, China; c Zhejiang Key Laboratory of Tumor Molecular Diagnosis and Individualized Medicine, Zhejiang Provincial People's Hospital, Affiliated People's Hospital, Hangzhou Medical College, Hangzhou, 310014, China; d Emergency Department, Tiantai People's Hospital of Zhejiang Province (Tiantai Branch of Zhejiang Provincial People's Hospital), Hangzhou Medical College, Taizhou, 317200, China; e Department of Emergency and Critical Care Medicine, Pudong New Area People's Hospital Affiliated to Shanghai Jiao Tong University School of Medicine, Shanghai, 201299, China; f Department of Surgical Oncology, Hangzhou Cancer Hospital, Hangzhou, 310002, China

**Keywords:** Oncolytic adenovirus, Cholesterol, Apolipoprotein E, Blood-brain barrier, Immunogenic cell death

## Abstract

Glioblastoma (GBM) remains a therapeutic challenge due to its aggressive behaviour and the limitation of drug delivery by the blood-brain barrier (BBB). Conventional oncolytic adenoviruses (OAs) suffer from poor targeting efficiency. To overcome this limitation, we developed a cholesterol-modified OA (OA@Cho). This engineered virus actively regulates protein corona formation in the bloodstream, selectively enriching apolipoprotein E (ApoE). By exploiting low-density lipoprotein receptor (LDLR)-mediated BBB transcytosis, OA@Cho achieves precise glioma targeting and enhances therapeutic delivery. Critically, upon reaching the GBM site, OA@Cho induces an anti-tumor immune response, turning "cold" tumors into "hot" tumors by inducing immunogenic cell death (ICD). The proposed "surface modification-ApoE enrichment-receptor-mediated" paradigm establishes a transformative platform that enables oncolytic viruses to bypass biological barriers, thereby advancing targeted viral therapies against CNS malignancies with high translational relevance.

## Graphical abstract

**Figure:**
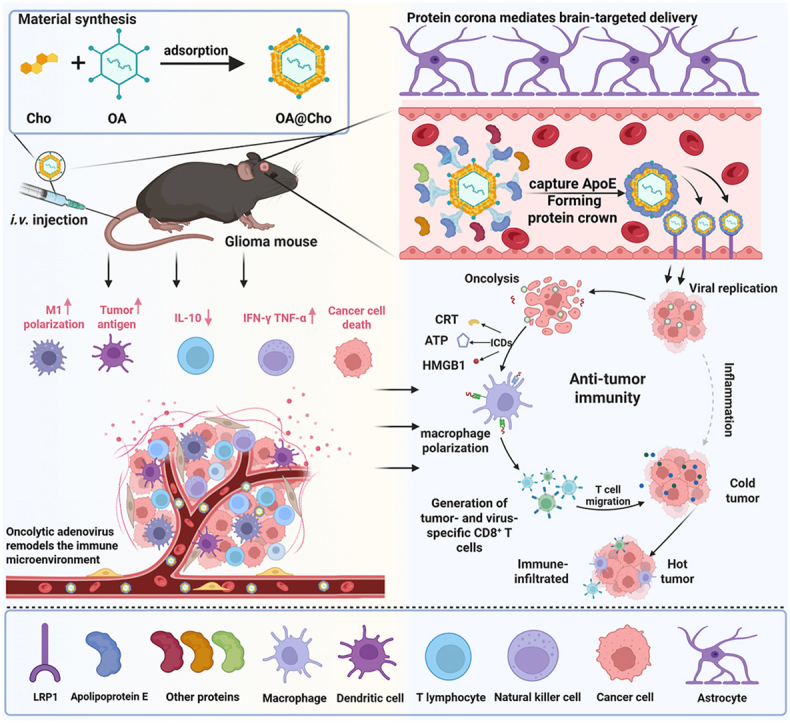

## 1 Introduction

GBM, as the most aggressive malignant tumor in the central nervous system, faces three core challenges in clinical treatment: significant intratumoral heterogeneity, diffuse infiltrative growth characteristics, and multiple drug resistance mechanisms [1,2]. According to the World Health Organization Classification of Central Nervous System Tumors (WHO CNS5, 2021), adult-type diffuse gliomas are now classified based on integrated histopathological and molecular features, with IDH-wildtype glioblastoma representing the most common and aggressive subtype [3]. Although the current standard treatment protocol based on surgery combined with radiotherapy provides short-term control of the disease, the median overall survival of patients is still limited to 14–16 months, with a five-year survival rate of less than 5 % [4,5].Immunotherapy is revolutionizing the treatment strategy of GBM: neo-antigenic vaccines achieve targeted killing through precise activation of tumor-specific T cells; immune checkpoint inhibitors break through the blood-brain barrier to remodel the immune microenvironment; and engineered oncolytic viruses create a dual killing effect by directly lysing tumor cells and releasing antigens to activate systemic immune responses [[6], [7], [8]]. OAs are genetically modified adenoviruses with the ability to selectively replicate in and lyse tumor cells [9]. Compared with conventional radiotherapy, oncolytic virotherapy has significant tumor-specific advantages and can achieve precision treatment in the context of increasing tumor heterogeneity and drug resistance issues [10,11]. Recent studies have shown that oncolytic viruses significantly activate the immune system by disrupting tumor cell membranes, releasing tumor antigens, and inducing local immune responses, thereby enhancing immune surveillance and suppressing tumor recurrence [12,13]. Therefore, oncolytic viruses are not only a single therapeutic tool, but also an innovative therapeutic strategy that integrates virology, immunology, and oncology. However, there are still many challenges in the practical application of oncolytic virotherapy: the clinical use of intratumoral drug delivery ensures a high concentration of the virus in the target area, but it may cause localized excessive immune activation (*e.g*., cerebral edema) and non-specific damage caused by the spread of the virus [[14], [15], [16]]. Therefore, how to reduce the damage of oncolytic viruses to normal tissues, improve their therapeutic efficacy and overcome immune escape is a hot topic of current research.

Systemic delivery systems for oncolytic virotherapy face a dual challenge: rapid clearance of exogenous viruses by the host immune system (particularly in the liver, spleen, and lungs), as well as the stringent limitations imposed on central delivery by the blood-brain barrier [17,18]. Studies have revealed that trans-BBB-tumor co-expressed receptors such as LDL receptor family and transferrin receptor can serve as targeting breakthroughs; however, the actual delivery efficiency remains significantly lower than the theoretical expectation [[19], [20], [21], [22]]. Therefore, the development of engineered carriers with both blood-brain barrier penetration and tumor-targeting capabilities remains an important research direction.

With the continuous advancement of targeting technology, nanotechnology, and immunomodulatory strategies, oncolytic virus therapy is expected to play a more important role in future cancer treatment [23,24].When nanoparticles enter the biological fluids, their surfaces form "protein corona" (PC) with plasma proteins, a process that irreversibly alters the physicochemical properties of the nanoparticles (e.g., hydrated particle size, dispersion stability, surface potential) and significantly impacts their targeting efficiency, biocompatibility, and in vivo metabolic behavior [[25], [26], [27], [28]]. Therefore, cells usually recognize nanoparticle-protein complexes rather than the nanoparticles alone. Targeting ligand-modified nanodrugs often lose their original targeting ability due to protein corona formation [29,30]. To address this issue, modulating the interaction force between nanomedicines and proteins to transform the "unfavorable" into "favorable" may provide new ideas for more precise drug delivery. In recent years, innovative strategies to overcome the BBB for GBM therapy have continued to emerge. For example, Wang et al. developed a class of fluorescent anticancer agents that not only penetrate the BBB effectively but also concurrently induce paraptosis and ferroptosis, demonstrating a promising chemical-based approach to activate non-apoptotic cell death pathways [31]. Building on such advances in small-molecule strategies—which benefit from efficient penetration—OAs offer a distinct multimodal platform that integrates direct tumor lysis with potent immunostimulation. Nevertheless, the systemic application of OAs remains challenging due to rapid clearance and inadequate BBB penetration.

As a key component of cell membranes, cholesterol participates not only in maintaining membrane microregion structure and signal transduction, but also exhibits abnormal metabolism closely linked to atherosclerosis, neurodegenerative diseases and malignant tumors [32,33]. LDLR and Low density lipoprotein receptor associated protein 1 (LRP1) are key receptors in the endocytic pathway, and their ligands include lipoprotein particles (such as LDL, VLDL) modified with apolipoprotein (such as ApoE), rather than free cholesterol [34,35]. In the blood, protein coronas adsorbed on the surface of nanocarriers may be rich in these lipoproteins [36], thereby mediating cellular uptake through LRP1/LDLR. However, cholesterol itself is not a direct ligand, but is transported as a core component of lipoproteins [37]. Studies have found that the protein coronas on nanoparticles circulating in the serum of hypercholesterolemic patients show apolipoprotein enrichment and a decrease in complement proteins, leading to an increased distribution of nanoparticles in the liver, spleen, and brain [38,39]. This phenomenon suggests that cholesterol plays a crucial role not only in lipoprotein metabolism but also in regulating the biodistribution and targeting ability of nanoparticles.

However, it is crucial to clarify that cholesterol itself is not a direct ligand for LDLR or LRP1 [34]. Instead, these receptors recognize specific apolipoproteins, most notably ApoE, which is present on the surface of lipoprotein particles such as LDL and VLDL [37]. Cholesterol is transported in the bloodstream primarily as a core component of these lipoproteins.

**Scheme 1:**
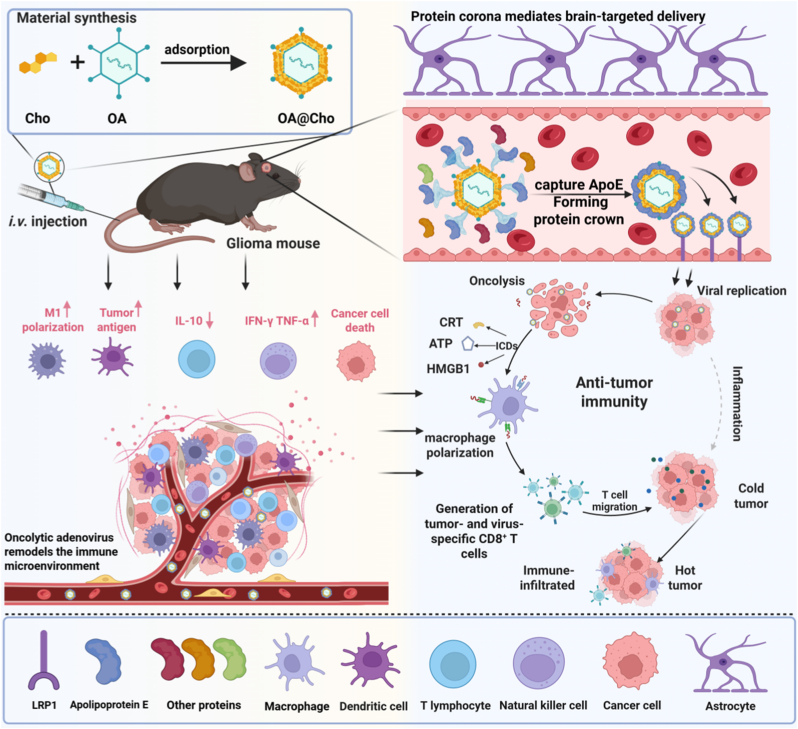
Schematic diagram of the mechanism of OA@Cho preparation and in vivo adsorption of ApoE to form a protein corona-mediated GBM immunotherapy. oncolytic adenovirus and cholesterol self-assembled into OA@Cho. Upon entering the blood environment after intravenous injection, OA@Cho then adsorbed more ApoE and effectively penetrated the blood-brain barrier and targeted GBM by using transcytosis mediated by the interaction of ApoE with the LRP1 receptor, which was highly expressed on the surface of gliomas, so as to allow oncolytic adenoviruses to enter into the brain in a greater number of ways. Within gliomas, OA@Cho was able to induce immunogenic cell death by releasing DAMPs. Simultaneously, activation of DCs and CD8^+^ T cells as well as polarization of M1 and conversion of immunosuppressive "Cold" GBM into immunoreactive "Hot" tumors enhanced anti-tumor immunity.

To enhance brain targeting, we designed OA@Cho. This design precisely modulates the composition and function of the formed protein corona to improve adenovirus delivery to the brain. Upon entering the circulation, OA@Cho binds to brain-targeted lipoproteins through cholesterol-lipoprotein lipid structural domain interactions. This allows the receptor-binding domain of the lipoprotein to be located on the surface of the virus, facilitating recognition by receptors (e.g., LDLR, LRP1) and promoting receptor-mediated transport across the blood-brain barrier. Thus, OA@Cho achieves efficient brain-targeted delivery, and intratumoral replication triggers a dual tumor killing mechanism: direct tumor lysis and ICD. The resulting local cytotoxicity synergizes with systemic immune activation to transform immunologically "cold" tumors into "hot" phenotypes, significantly enhancing anti-tumor immunity (Scheme 1).

## 2 Materials and methods

### 2.1 Materials

Oncolytic adenovirus (H101), an engineered virus based on human type 5 adenovirus with a deleted E1B-55kD gene and a 78.3–85.8 mu fragment in the E3 region, was obtained from Shanghai Sanwei Biotechnology Co., Ltd. Fetal bovine serum (FBS) was purchased from Hangzhou Yangming Biotechnology Co, Ltd. Cy5 NHS coupling dye and Calcein/PI cell activity detection reagent were purchased from sigma Aldrich (USA). cholesterol (c8667) was purchased from sigma Aldrich. Anti-ApoE (ab183597), anti-transferrin receptor (ab214039) and anti-adenovirus type 5 hexon (ab316852) primary antibody were purchased from Abcam (UK). Recombinant human ApoE protein (paa704hu01) was purchased from cloud clone (Wuhan, China). The total cholesterol assay kit (bc1980) is from Solarbio. The mouse ApoE ELISA kit (JL12945) was purchased from Shanghai Jonlnbio Industrial Co., Ltd.. Total cholesterol determination kit (bc1980), Probucol (S31624) was purchased from Shanghai yuanye Bio-Technology. BCA protein quantitative kit, annexin V-FITC apoptosis detection kit and hoechst33342 nuclear dye were purchased from beyotime Biotechnology Co., Ltd. (Shanghai, China).

### 2.2 Preparation and characterization of OA@Cho

OA (10 μL, 1 × 10^12^ viral particles (VPs)) and cholesterol (50 μL, 3 mmol/L) underwent overnight incubation in PBS at 4 °C. The mixture was then subjected to aseptic filtration (0.22 μm membrane) and stored at 4 °C. The morphology of oncolytic viruses was observed using a transmission electron microscope (HT7800, Hitachi, Japan). Hydration particle size, zeta potential and polydispersity index (PDI) were measured using a dynamic light scatterometer (Zetasizer nano zs90, Malvern, UK), OA particle size distribution was measured with a NanoCoulter counter (Resun Technology, Co., Ltd., Shenzhen).

### 2.3 Preparation and purification of protein corona

OA or OA@Cho (10^8^ PFU/mL) was incubated with mouse plasma (100 μL, 37 °C, 2 h). The protein corona-NP complexes were pelleted by centrifugation (20,000 *g*, 4 °C, 2 h), washed 3× with PBS (20,000 *g*, 10 min), and resuspended in PBS. Protein corona was quantified via BCA assay.

### 2.4 Detection of the affinity of ApoE to virus cholesterol nanoparticles by Microscale thermophoresis (MST) method

The equilibrium dissociation constant (KD) of ApoE was determined by MST method (NanoTemper Technologies). First, the protein was mixed with ApoE and dye at a 1:1 ratio, incubate at room temperature for 30 min, centrifuge the sample at 15000 *g* at 4 °C for 10 min, and take the supernatant into a new tube. Then OA and OA containing 10^8^ pfu of the same number of virus particles were prepared OA@Cho and then mixed with the same volume of ApoE solution (200 nM). Next, the series of mixtures were pulled into the capillaries and detected by 40 % power. The direct binding of ApoE to oncolytic adenovirus is reflected in the changes of thermal electrophoresis of fluorescent conjugated protein during the formation of complex. The data were analyzed by Mo affinity analysis software (version 2.3).

### 2.5 Dot blotting

OA or OA@Cho (1 × 10^10^ VPs each) were spotted onto the NC membrane (2 μL per spot; Solarbio). The membranes were incubated overnight at 4 °C with a recombinant anti-adenovirus 5 hexon antibody (ab316852; 1:1000 dilution) and then rinsed three times with TBST for 10 min each. After incubation with HRP-conjugated secondary antibody (Huabiotech; 1:5000, PBS) for 2 h at RT and similar washes, the membranes were treated with HRP-conjugated anti-rabbit IgG (CST, 7077/7076). The immunoreactive bands were visualized using an enhanced chemiluminescence (ECL) substrate and imaged with a ChemiScope Series Imaging System (qTouch，RWD, Shenzhen, China).

### 2.6 Wound healing test

GL261 cells (1 × 10^5^ cells/well^)^ were seeded a 6-well plate and culture until fusion. A scratch model was created in the middle of the culture dish with the tip of a 200 μL pipette. Then, GL261 cells were treated with PBS, Cho, OA, OA@Cho, OA-ApoE, or OA@Cho-ApoE, and the cell images were observed under a microscope after 24 h of culture. Finally, the relative migration distance of the cells was calculated by ImageJ software.

### 2.7 In vitro blood brain barrier model and permeability study

To study the ability of OA@Cho-ApoE to penetrate the BBB, we constructed an in vitro blood-brain barrier model. bEnd.3 cells (1.0 × 10^5^ cells/well) were seeded onto the upper chamber of a Transwell insert (0.4 μm polyester membrane) placed in a 12-well plate. When the transepithelial electrical resistance (TEER) value exceeded 200 Ω cm^2^, the lower chamber medium was aspirated and GL261 cells (4.0 × 10^5^ cells/well) were inoculated into the lower chamber medium. After 8 h of incubation with the drug treatment, GL261 cells in the lateral basolateral chamber were collected, and the fluorescence penetrating into the lower chamber was detected by flow cytometry, confocal laser scanning microscopy (CLSM).

### 2.8 Cellular uptake

To assess nanoparticle uptake by GL261 cells, cells were seeded in confocal dishes (2 × 10^5^ cells per well) and treated with OA or OA@Cho-ApoE for varying durations. After fixation, nuclei were stained with Hoechst 33342 (30 min), followed by three PBS washes. Uptake was visualized by CLSM. Images from both the apical and basolateral chambers were captured using a system configured with the appropriate filters for Cy5 fluorescence.

### 2.9 Construction of a Luc^+^-GL261 orthotopic glioma model

Female C57BL/6 mice (6–8 weeks old) were purchased from Hangzhou Qizhen Laboratory Animal Science and Technology Co. Ltd. To construct the C57BL/6 mouse brain tumor model, mice were placed under gas anesthesia and fixed on a brain stereotaxic apparatus. Following craniotomy, 2 μL of PBS suspension containing 5.0 × 10^4^ Luc^+^ -GL261 cells was stereotactically injected into the right striatum using a Hamilton microsyringe (Model 700 Series KH, needle gauge 26s) at the following coordinates relative to bregma: 0.5 mm posterior, 2.0 mm lateral right, 2.5 mm ventral. An in vivo imaging system (IVIS) was used to monitor tumor growth in the brain by bioluminescent signals.

### 2.10 Monitoring the biodistribution of drugs

After validation of a successful orthotopic glioma mouse model, a 100 μL dose of free ICG-NHS, OA, or OA@Cho (1 × 10^10^ VPs) was injected. Biodistribution was recorded noninvasively at 2, 4, 8, 12, and 24 h using IVIS imaging. Euthanasia was performed 8 h after injection, followed by collection of major organs and tumor tissue. in vivo organ distribution was assessed by IVIS imaging. Tumor tissues were sectioned, DAPI stained, and ICG fluorescence was detected using CLSM. Quantitative analysis of fluorescence intensity in the tissues provided data on in vivo distribution and tumor targeting efficiency.

### 2.11 Photoacoustic imaging (PAI) in vivo

The distribution of OA and OA@Cho (100 μL, 1 × 10^10^ VPs) in nude mice was assessed using in vivo photoacoustic imaging (LOIS-3D system, Tomowave, USA). Under isoflurane anesthesia, the drugs were injected and PAI was performed at 880 nm after an interval of 8 h. The mice were then euthanized, and brain tissues were collected for further examination.

### 2.12 Antitumor efficacy in vivo

All animal experiments were conducted and approved by the Animal Ethics Committee of Zhejiang Provincial People's Hospital. One week after Luc^+^- GL261 tumor cells were inoculated, all mice were randomly divided into four groups (n = 6), (1) control (PBS treatment); (2) Cho; (3) OA; (4) OA@Cho One dose was given on the 8th, 10th, 12th, 14th and 16th day respectively. To monitor the inhibitory effect of different drugs on glioma, fluorescein potassium (10 μL/g) was injected intraperitoneally on the 7th, 10th, 13th, 16th and 19th day after implantation, respectively. The bioluminescence intensity of tumor was dynamically observed by IVIS imaging. After unifying the relative photon flux of the tumor, the fluorescence intensity curve was drawn. The calculation formula of tumor inhibition rate in each group: tumor inhibition rate (TIR) = (I_PBS_- I_therapy_)/I_PBS_ × 100 %, in which I_PBS_ is the bioluminescence intensity of brain tumor on the day of PBS group, and I_therapy_ is the nano drug treatment group. At the same time, the tumor bioluminescence growth rate before and after treatment was calculated = I_Day19_/I_Day7_. During the treatment, the weight of mice was recorded every 2 days, and the Kaplan Meier survival curve was generated.

### 2.13 Histological and safety analysis

Mice were euthanized on day 19 after drug administration, and their organs and brains were collected. Tissues were fixed in 4 % paraformaldehyde, embedded in paraffin, sectioned, and stained with H&E. Histopathological analysis was performed using immunofluorescence (IF) and immunohistochemistry (IHC). Tumor proliferation and apoptosis were assessed via Ki67 IHC and TUNEL staining, respectively. IF sections were imaged using a fluorescence microscope. IHC results were observed with a light microscope. Furthermore, we evaluated the expression of CRT, HMGB1, CD3/CD8, Foxp3/CD4, and CD206/CD86 by IF. By obtaining the serum of tumor-bearing mice after treatment, the in vivo safety of oncolytic adenovirus was studied to evaluate liver function (AST, ALT, TP, ALP, ALB), renal function (CREA, BUN, TBIL, UA), myocardial enzyme spectrum (LDH) and blood lipid (TC). By obtaining the serum of glioma-bearing mice before and after treatment, the effect of oncolytic adenovirus on lipoproteins in vivo was analyzed.

### 2.14 Measurement of cytokines

To study changes in cytokines, blood samples were collected from glioma mice after different treatments. Serum levels of cytokines TNF-α, IFN-γ, IL-1, IL-6 and IL-10 were measured and quantified using ELISA kits (JONLNBIO, China).

### 2.15 Cytometry by Time-Of-Flight (CyTOF)

The antibodies for mouse tumor immunoassay of 27 indicators (PMRP17) and other reagents used in the CyTOF experiment were all purchased from Polarisbiology. For the preparation of single-cell suspensions of GBM tissues, mice were anesthetized and sacrificed, and the brains were harvested and sectioned into small pieces in RPMI-1640 medium with 1 mg/mL collagenase IV (BioFroxx) and 0.1 mg/mL DNase I (BioSharp). The tissue pieces were digested for 30 min and grinded through 70 μm nylon filters (Biologix). Red blood cell lysis buffer was added and washed. Then, 30 % percoll solution was added. After centrifugation, floating white myelin tissue could be seen in the upper layer, and cell precipitation in the lower layer. The supernatant and myelin debris were gently aspirated, and the cell pellet was washed with cell staining buffer. Next, the cells were stained with the cisplatin solution. The mixture of 27 antibodies was added to each tube after the closure of the Fc receptor. After staining with Ir-DNA chimeric reagent, the samples were suspended and collected by Lumarion R-XC Mass Cytometry System (Polaris Biology Co., Ltd.).

### 2.16 Statistical analysis

All data were statistically analyzed using GraphPad Prism 10.0 software. Data are expressed as mean ± standard deviation (SD). Comparisons between two groups were made using the unpaired *t*-test, and comparisons between multiple groups were made using two-way ANOVA. The significance was ∗*p* < 0.05,∗∗*p* < 0.01,∗∗∗*p* < 0.001.

## 3 Results

### 3.1 Synthesis and characterization of OA@Cho

**Fig. 1:**
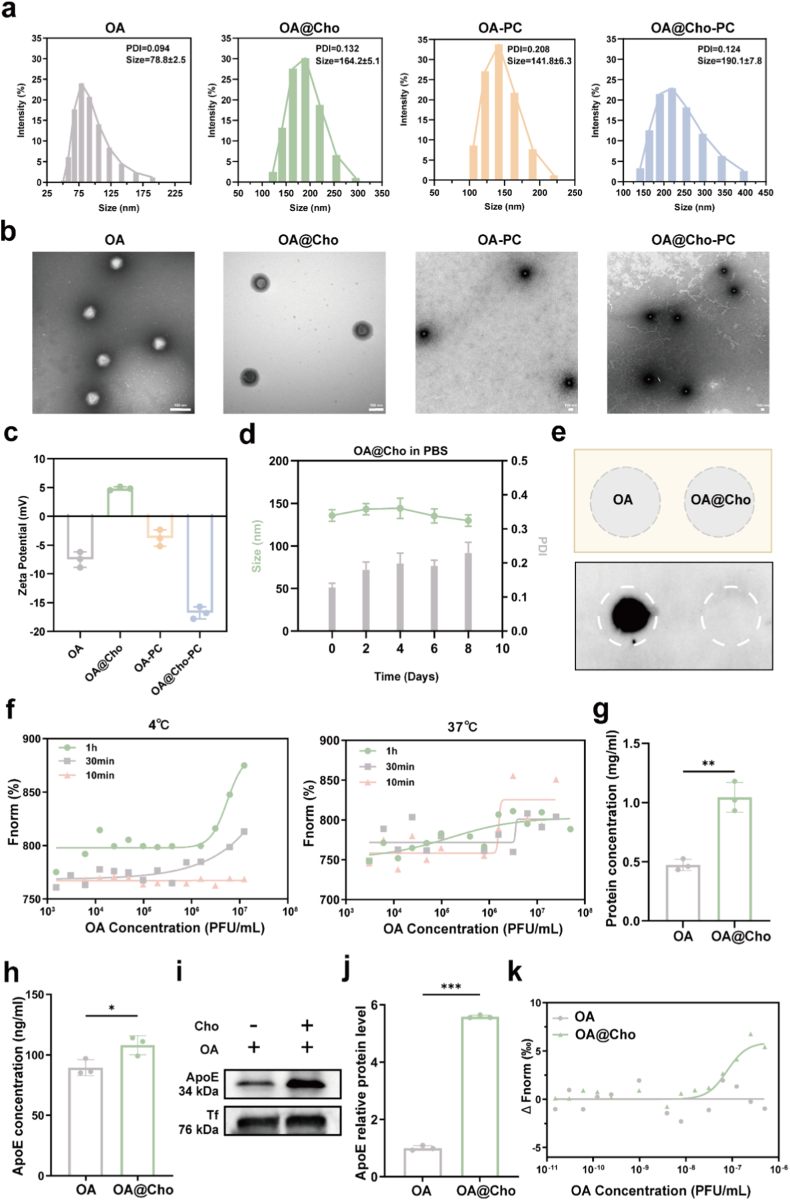
Preparation and characterization of OA@Cho. a) Hydrodynamic diameters of OA, OA@Cho, OA-PC, and OA@Cho-PC NPs. b) TEM images of OA, OA@Cho, OA-PC, and OA@Cho-PC NPs. Scale bar: 100 nm. c) Zeta potentials of OA, OA@Cho, OA-PC, and OA@Cho-PC NPs. d) Hydrodynamic diameter and PDI of OA@Cho nanoparticles incubated in PBS at 37 °C over a period of 8 days. e) Dot blotting of OA and OA@Cho. f) Binding affinity of cholesterol for OA at the equilibrium temperature of 4 °C (37 °C) for different time conditions. g) Total protein content of OA and OA@Cho forming protein coronas. h) ApoE content in protein coronas formed by OA and OA@Cho. i, j) Western blotting of ApoE adsorbed on OA and OA@Cho. k) The binding affinity of ApoE for OA in the presence of cholesterol and the binding affinity of APOE for naked OA were examined by MST. Data are presented as mean ± SD. (n = 3) Two-way ANOVA were used to compare whether there are significant differences between data.

In this study, the surface of OA particles was enriched with carboxyl residues, conferring a negative surface charge under physiological pH conditions, thus allowing surface modification using cholesterol. Like other nanoparticles, OA particles form a PC in plasma (Fig. S1a). Dynamic light scattering (DLS) characterization showed that cholesterol modification increased the size of the hydrated OA particles from 78.8 nm to 164.2 nm, suggesting a "cholesterol-coupled OA complex" (OA@Cho). Notably, in the simulated physiological fluid environment, the corona diameter of proteins formed on the bare OA surface increased to 141.8 nm, whereas the corona diameter of proteins formed by OA@Cho further increased to 190.1 nm (Fig. 1a; Fig. S1b), suggesting that cholesterol modification enhances the protein adsorption capacity of the virus. Nanoparticle tracking analysis (NTA) confirmed an OA particle size distribution centered around 80 nm, with uniform dispersion and a total viral particle concentration of 10^10^ PFU/mL (Fig. S1c). Transmission electron microscopy (TEM) characterization showed that both naked OA and OA@Cho exhibited good monodispersity, with visible cholesterol adsorption on the OA@Cho surface. Notably, both OA and OA@Cho surfaces were surrounded by a protein corona resembling a "halo". (Fig. 1b). In addition, zeta potential analysis showed that cholesterol modification significantly increased the surface charge of OA, again confirming the successful adsorption of cholesterol to the viral surface. The zeta potentials of both OA-PC and OA@Cho-PC shifted after the formation of protein corona (Fig. 1c). The stronger negative charge on the surface of OA@Cho-PC may have originated from the selective adsorption of anionic apolipoproteins. To further evaluate the colloidal stability of OA@Cho, we incubated OA@Cho in phosphate-buffered saline (PBS), complete cell culture medium (DMEM supplemented with 10 % fetal bovine serum, FBS), and artificial cerebrospinal fluid (aCSF) at 37 °C. The hydrodynamic diameter and PDI were monitored over 8 days by DLS. OA@Cho maintained excellent stability in all tested media without significant aggregation or increase in PDI (Fig. 1d; Fig. S1d–f). This comprehensive stability profile confirms the robustness of OA@Cho for applications in complex biological environments, including systemic circulation and the central nervous system. Dot blotting confirmed the presence of effectively modified virus particles (Fig. 1e). MST analysis further verified the stable binding of OA to cholesterol at across temperatures (4 and 37 °C), indicating strong thermodynamic interactions (Fig. 1f; Fig. S1g). These comprehensive physicochemical characterizations confirm the successful development of cholesterol-modified OA nanodelivery systems exhibiting enhanced stability and modified surface properties, laying a solid foundation for subsequent biomedical applications.

ApoE is an important member of the lipoprotein superfamily, and as a multifunctional lipid transporter protein, it plays a central role in systemic cholesterol metabolism and lipid homeostasis regulation [40]. Numerous clinical studies have shown that ApoE gene polymorphisms (especially ApoE4 isoforms) are significantly associated with the pathologic course of Alzheimer's disease and the risk of cardiovascular disease [40,41]. In the physiological state, ApoE is involved in the dynamic homeostatic regulation of lipid metabolism mainly through signaling pathways mediated by the LDLR and its related receptor family. Notably, as the predominant cholesterol transport carrier in the central nervous system, ApoE plays an irreplaceable physiological role in maintaining blood-brain barrier integrity, synaptic plasticity, and neuronal function [42,43]. This study found that serum ApoE levels were significantly elevated in glioma model mice compared to normal mice (Fig. S1h), creating a favorable pathological microenvironment for OA@Cho nanoparticle targeting. Subsequently, the total protein content was adsorbed in OA@Cho Incubated with glioma mouse plasma at 4 °C for 1 h, detected after ultracentrifugation, and analyzed by Coomassie brilliant blue staining, the results showed that the total protein content adsorbed on the surface of OA@Cho was significantly higher than that of the bare OA group (Fig. 1g; Fig. S1i).

In response to the question of whether more ApoE would be adsorbed, we tested the content of ApoE in the total proteins on the surface after ultracentrifugation, and found that higher ApoE content on OA@Cho was also higher than that of the OA group (Fig. 1h). Further by protein blotting analysis, we systematically compared the adsorption capacity of bare OA and OA@Cho for ApoE. The results showed that higher ApoE levels in the total protein adsorbed onto OA@Cho was significantly higher than that of the bare OA group, indicating that cholesterol modification significantly enhanced the specific adsorption capacity of the nanoparticles for ApoE (Fig. 1i and j). To further elucidate the interaction mechanism between ApoE and nanoparticles, we employed MST to quantitatively analyze their binding characteristics. The results showed that the binding constant of OA@Cho to ApoE was significantly lower than that of bare OA, indicating that the cholesterol modification resulted in a significant increase in the affinity of the nanoparticles for ApoE (Fig. 1k). Binding free energy calculations further confirmed that cholesterol modification significantly enhanced the thermodynamic interactions between ApoE and the nanoparticles. To comprehensively evaluate the composition of the protein corona and address the potential impact of other components, we conducted a study on OA and OA@Cho After incubation in the plasma of glioma mice. The results confirm that ApoE is OA@Cho (Fig. S1j and k) adsorbs more than OA. It is worth noting that OA@Cho compared with naked OA, the abundance of several conditioners has also decreased. The change in protein corona distribution - enhancing targeting ligands while reducing immunogenic signals - will improve circulation in the blood and targeting efficiency in the brain. This discovery establishes a biomimetic targeting paradigm leveraging endogenous lipoprotein trafficking for designing novel targeted nanodelivery systems and offers new insights into targeted therapeutic strategies for diseases such as glioma.

### 3.2 Antitumor effect in vitro

**Fig. 2:**
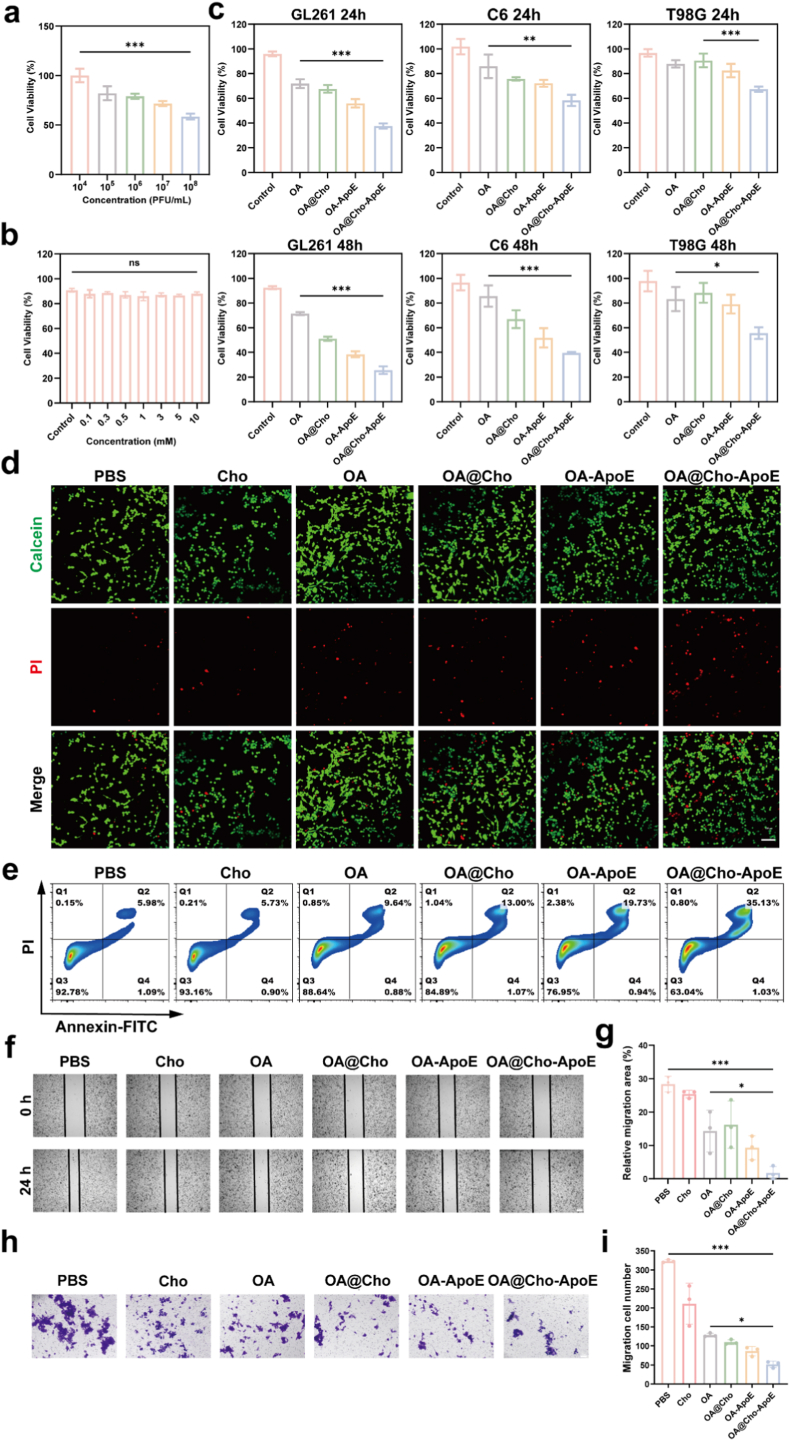
In vitro therapeutic performance of OA@Cho. a) Effect of oncolytic adenovirus on cell ability at different PFU/mL. b) Effects of cholesterol on cell ability. c) Effect of OA@Cho-ApoE on cell ability of different glioma cell lines. d) GL261 cells were treated with PBS, Cho, OA, OA@Cho, OA-ApoE, OA@Cho-ApoE for 24 h, and Calcein-AM (green) and PI (red) were used for live-dead staining of cells. Scale bar: 100 μm. e) PBS, Cho, OA, OA@Cho, OA-ApoE, or OA@Cho-ApoE. After 24 h of treatment, the representative flow diagrams of GL261 cells stained with annexin V-FITC and PI were obtained. f, g) Inhibition migration of GL261 cells was examined by wound healing assay after 24 h and quantification data. Scale bar: 200 μm. h, i) Transwell assay of GL261 cells after OA@Cho NPs after 24 h and quantification data. Scale bar:100 μm. Data are presented as mean ± SD. (n = 3) two-way ANOVA were used to compare whether there are significant differences between data. (For interpretation of the references to colour in this figure legend, the reader is referred to the Web version of this article.)

Based on the previous systematic studies on the characterization of the physicochemical properties of OA@Cho nanocomplexes and their ApoE adsorption capacity, we further evaluated the antitumor effects of this nanosystem in vitro. First, the cytotoxicity of OA at different multiplicities of infection (MOI) on GL261 glioma cells and bEnd.3 normal cells at different time points (24 h and 48 h) was examined by cell counting kit-8 (CCK-8) method. The results showed that at a concentration of 10^8^ PFU/mL (plaque-forming units per milliliter), OA exhibited a significant killing effect on GL261 cells (Fig. 2a), whereas there was no significant toxicity on bend.3 normal cells (Fig. S2a and b). Therefore, all subsequent experiments were performed using this viral titer. Considering the possible effects of cholesterol modification on cell activity, we evaluated the toxicity of different concentrations of cholesterol (0–10 mM) on GL261 and bEnd.3 cells. The results showed that there was no significant difference in cell activity in the cholesterol-treated group compared to the control group within the experimental concentration range, indicating that the cholesterol concentration used did not have a significant effect on cell growth (Fig. 2b; Fig. S2c and d). In addition, when simulating the adsorption of ApoE in vitro and in vivo, considering whether ApoE would be toxic to GL261 cells, the results showed that ApoE (0–5 mM) was not toxic to cells (Fig. S2e). Subsequently we treated GL261, C6, T98G glioma cell lines at 24,48 h using different groups of drugs and found that the cell survival rates were at 37.5 %, 58.3 %, 67.4 % after 24 h of treatment with OA@Cho-ApoE complex and 25.7 %, 39.6 %, 55.6 % after 48h of treatment, respectively (Fig. 2c). We have noticed that the following drugs have different cytotoxic effects OA@Cho-ApoE Crossing glioma cell lines. To investigate whether this variability is related to the expression of target receptors, we analyzed the LDLR protein levels through Western blotting. The results confirmed that GL261 cells expressed slightly higher levels of LDLR compared to T98G cells (Fig. S2f), providing a mechanistic explanation for cell type dependent therapeutic efficacy.

To investigate the anti-tumor mechanism of OA@Cho-ApoE complex, we first examined its effect on the energy metabolism of tumor cells. ATP content measurement showed that after 6 h of OA@Cho-ApoE treatment, the intracellular ATP level of GL261 cells was significantly reduced to 29.3 % of that of the control group (Fig. S2g). It was further confirmed by live-dead cell staining experiments that the mortality rate of GL261 cells was significantly higher than that of other treatment groups after 24 h of OA@Cho-ApoE treatment, suggesting that ApoE-mediated targeting significantly enhanced the anti-tumor effect of the nanocomplexes (Fig. 2d). Western blot analysis suggested a trend wherein OA@Cho-ApoE treatment appeared to increase the expression of the pro-apoptotic protein Bax and decrease the expression of the anti-apoptotic protein Bcl-2. we observed a statistically upregulation of Caspase-9. (Fig. S2h–k). The Annexin V-FITC/PI double staining assay further confirmed that the apoptosis rate in the OA@Cho-ApoE treatment group was significantly increased to 36.16 %, which was markedly higher than that in the PBS group, Cho group, OA group, OA@Cho group, and OA-ApoE group (Fig. 2e). These results were highly consistent with the results of CCK-8 experiments. In addition, the results of cell scratch assay and Transwell migration assay showed that the migration ability of GL261 cells was significantly inhibited after 24 h of OA@Cho-ApoE treatment, and the migration rate was reduced to 1.71 % and 15.9 % of the control group, respectively (Fig. 2f–i). The above experimental results fully confirmed that OA@Cho could effectively inhibit the proliferation of glioma cells, induce apoptosis and inhibit cell migration through ApoE-mediated targeted delivery system, which showed good prospects for antitumor applications.

### 3.3 Cellular uptake and blood brain barrier penetration in vitro

**Fig. 3:**
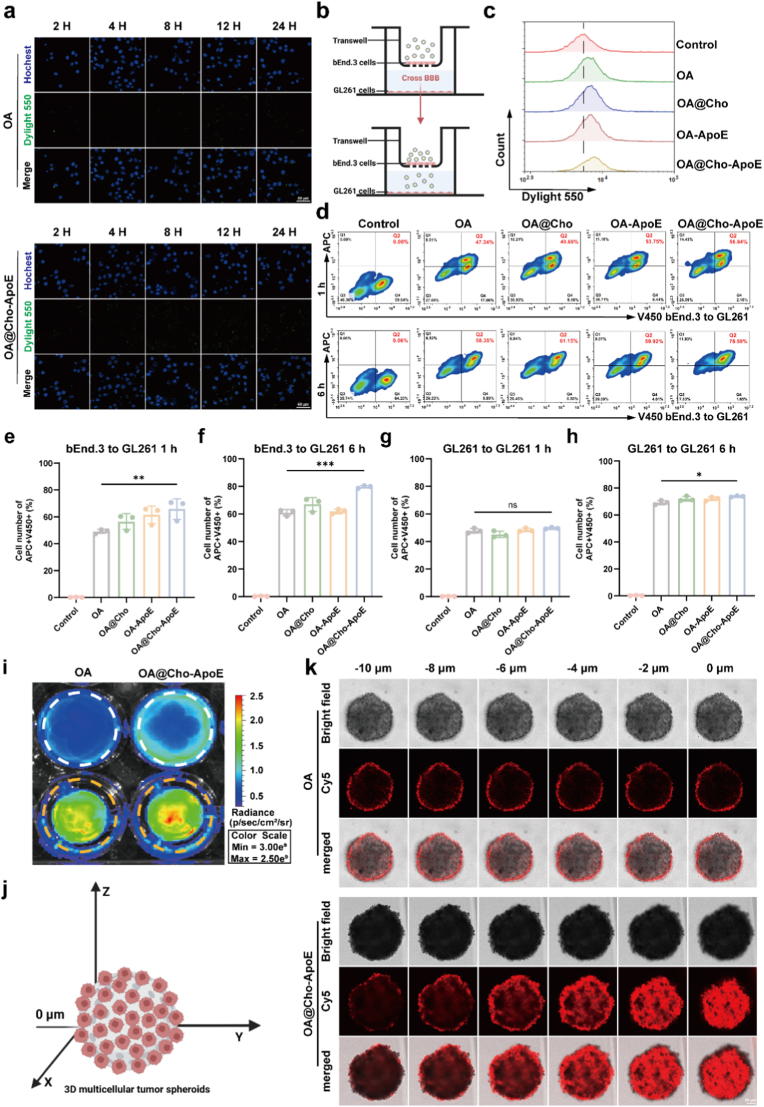
In vitro targeting of gliomas by OA@Cho adsorbed ApoE. a) CLSM images of GL261 cells incubated with OA and OA@Cho-ApoE at different time points. b) Schematic diagram of constructing the blood-brain barrier in vitro. Scale bar: 50 μm. c) Flow cytometry analysis of BBB penetration ability of GL261 cells treated with PBS, OA, OA@Cho, OA-ApoE and OA@Cho-ApoE for 8h. d) Flow cytometry was used to determine the transcytosis efficiency of OA, OA@Cho, OA-ApoE, and OA@Cho-ApoE into GL261 cells after 1 h and 6 h of bEnd.3 cells culture. e-h) Transcytosis efficiency of OA, OA@Cho, OA-ApoE and OA@Cho-ApoE from bEnd.3 to GL261 cells or from GL261 to GL261 cells at different times. i) Fluorescence images of transwell inserts and petri dish bottoms after incubation with different nanoparticles (White circle: lower chamber, yellow circle: upper chamber). j) Schematic illustration of 3D multicellular tumor spheroids. k) Tumor penetration ability of OA and OA@Cho-ApoE and after 8h of co-culture. Scale bar: 50 μm. Data are presented as mean ± SD (n = 3). two-way ANOVA were used to compare whether there are significant differences between data. (For interpretation of the references to colour in this figure legend, the reader is referred to the Web version of this article.)

**Fig. 4:**
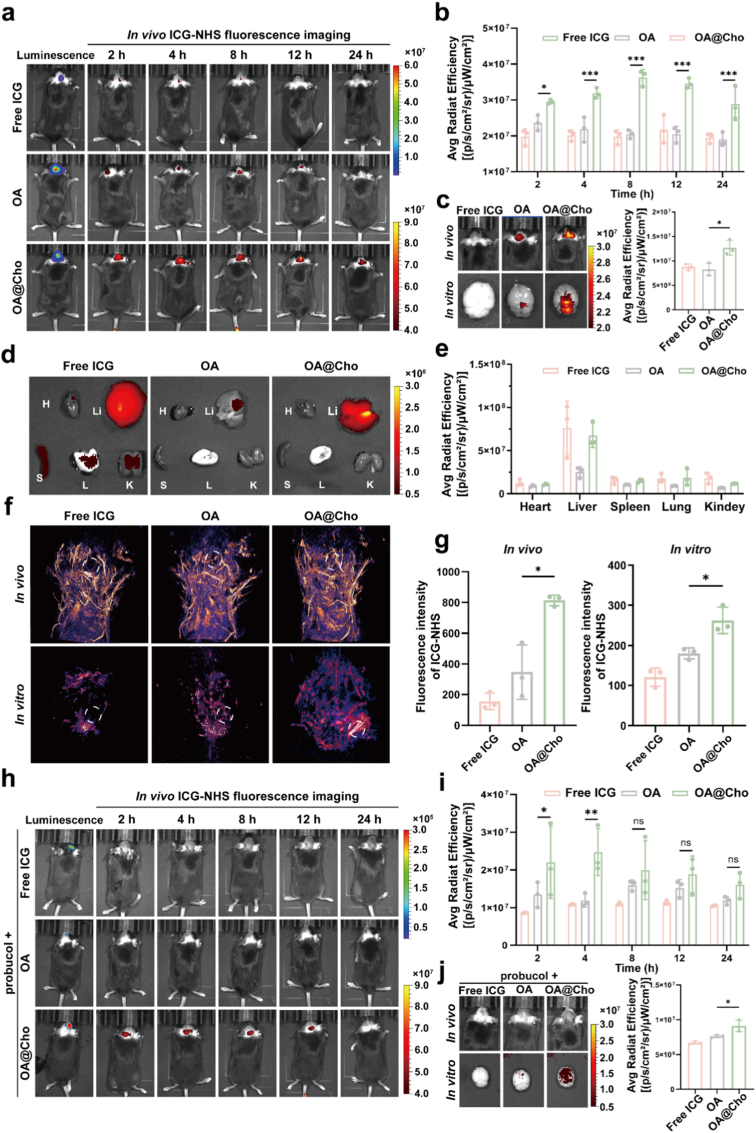
OA@Cho in vivo glioma and PAI capacity.a, b) Time-course in vivo fluorescence imaging (a) and quantitative analysis (b) of orthotopic glioma models following intravenous administration of free ICG, OA, and OA@Cho. c) In vivo Brain fluorescence and in vitro brain fluorescence intensity and fluorescence intensity statistics. d,e) Fluorescence images and quantitative analysis of heart, liver, spleen, lungs and kidneys after administration of Free, OA and OA@Cho for 8h. f,g) Live brain photoacoustic imaging and in vitro brain photoacoustic imaging and intensity statistics at 8 h. h,i)Fluorescence imaging and fluorescence intensity statistics of the brain of mice treated with Probucol at different time points. j) In vivo Brain fluorescence and in vitro brain fluorescence intensity and fluorescence intensity statistics with Probucol at 8 h. Data are presented as mean ± SD (n = 3). A *t*-test or two-way ANOVA were used to compare whether there are significant differences between data.

To evaluate the uptake efficiency of nanoparticles by GL261 cells, we first fluorescently conjugated the virus particles with DyLight^TM^ 550 NHS ester and removed the free dye by dialysis. The conjugated virus particles were co-incubated with GL261 cells, and the intracellular fluorescence intensity was observed by CLSM at 0, 2, 4, 8, 12 and 24 h time points, respectively. The results showed that the OA@Cho-ApoE-treated group exhibited the strongest intracellular fluorescence signal at 8 h, and its fluorescence intensity was significantly higher than that of the non-targeted OA-treated group (Fig. 3a; Fig. S3a). This phenomenon suggests that OA@Cho significantly increased the uptake efficiency of virus particles by tumor cells through the ApoE-mediated endocytosis pathway by leveraging the high expression of low-density lipoprotein LRP1, low-density lipoprotein receptor-associated protein 2 (LRP2), and LDLR on glioma cell membranes. The BBB, a highly selective semi-permeable boundary formed by endothelial cells, severely restricts drug delivery from circulation to the brain, contributing to poor glioblastoma treatment outcomes [31]. To address this, an in vitro BBB model was established using bEnd.3 cells. When the trans-endothelial TEER value was stabilized above 200 Ω-cm^2^ (Fig. S3b), it indicated that the BBB model was successfully constructed (Fig. 3b). By comparing the BBB penetration efficiencies of the four preparations, OA, OA@Cho, OA-ApoE and OA@Cho-ApoE, it was found that the transcytosis efficiency in the lower chamber was significantly higher in the OA@Cho-ApoE-treated group than in the other groups (Fig. 3c). Flow cytometry with Annexin V-FITC/PI staining revealed that OA@Cho-ApoE induced 22.58 % apoptosis in GL261 cells, significantly higher than untreated controls and other formulations, which was significantly higher than that of other treated groups, a result that functionally verified that OA@Cho-ApoE had a stronger BBB penetration ability (Fig. S3c). We further assessed OA@Cho-ApoE transcytosis from bEnd.3 to GL261 cells or between GL261 cells. Samples were pretreated with bEnd.3 or GL261 cells for 4 h before adding Hoechst-33342-stained GL261 cells for various durations (Fig. S3d). The OA group exhibited negligible transcytosis, as fluorescence showed little change over time. Notably, OA@Cho-ApoE presented an enhanced transcytosis effect over extended incubation periods (Fig. 3d–f; Fig. S3e). After 6 h of incubation, compared to OA, the transcytosis of OA@Cho-ApoE reached 78.59 % and 73.15 % (bEnd.3→GL261) and (GL261→GL261) (Fig. 4g and h; Fig. S3f). Fluorescence imaging results showed that the OA@Cho-ApoE-treated group exhibited stronger fluorescence signals in both the insertion and bottom chambers, further confirming its excellent BBB penetration performance (Fig. 3i).

To further evaluate the penetration ability of nanoparticles in solid tumors, we established a three-dimensional multicellular tumor spheroid (MCTS) model of GL261 cell origin (Fig. 3j). CLSM Z-stack imaging results showed that significant Cy5 fluorescence signals were observed at all scanning depths (especially at 0 μm) after 8 h of OA@Cho-ApoE treatment, whereas the untargeted OA-treated group only showed weak fluorescence in superficial areas (Fig. 3k). These results fully confirmed that OA@Cho-ApoE was able to effectively penetrate the BBB and reach deep into the tumor tissues, which was mainly attributed to its specific interaction with the LDLR family of receptors, which are highly expressed on the surface of BBB endothelial cells and tumor cells, via ApoE.

To confirm the important role of LRP1/LDLR in the uptake mechanism, we conducted receptor blockade experiments. Pre incubation of GL261 cells with anti-LRP1/LDLR blocking antibodies and anti-TfR blocking antibodies significantly reduced the internalization of GL261 cells OA@Cho-ApoE quantify by flow cytometry (Fig. S3g). This result directly supports that LRP1/LDLR mediated endocytosis is the main pathway to enhance the targeting and permeability of our nanoplatform.

### 3.4 In vivo glioma targeting and biodistribution

Based on the excellent BBB penetration ability and tumor-targeting properties demonstrated by OA@Cho in vitro experiments, we further evaluated their biodistribution and behavior in vivo. After constructing a Luc^+^-GL261 orthotopic glioma mouse model and verifying the successful establishment of the model using an IVIS, we carried out a systematic in vivo distribution study. OA and OA@Cho conjugated with ICG-NHS were intravenously injected into tumor-bearing mice. In vivo imaging revealed significantly stronger tumor fluorescence in the OA@Cho group versus OA at all time points, demonstrating enhanced OA targeting via cholesterol modification. Notably, the fluorescence signals of the OA@Cho-treated group remained strong at 24 h, while those of the OA group had largely disappeared, suggesting a longer in vivo circulation time for OA@Cho (Fig. 4a). Analysis revealed significantly greater tumor accumulation of OA@Cho compared to Free ICG and OA at 8 h post-injection (Fig. 4b), primarily due to its immune evasion and ApoE-mediated targeting. To clarify whether the tumor-targeting property of OA@Cho originated from ApoE adsorption, we compared the fluorescence distribution of the Free ICG, OA and OA@Cho groups after 8 h of treatment. The results showed that only the OA@Cho-treated group presented significant fluorescence signals in the brain tumor region, while no obvious tumor targeting was seen in the Free ICG group, ruling out the possibility of ICG self-targeting (Fig. 4c). Organ distribution studies showed that OA@Cho presented strong fluorescent signals in the liver and kidney, indicating that it was metabolically cleared mainly through the hepatic-renal pathway, which was consistent with the mechanism of nanoparticle capture and clearance by the reticuloendothelial system (Fig. 4d and e). Fluorescence imaging of brain tissue sections further confirmed that OA@Cho could still accumulate specifically at the tumor site 8 h after injection, while it was less distributed in normal brain tissue (Fig. S4a). In addition, we evaluated the PAI performance of OA@Cho for the first time. In vivo PAI results showed significant photoacoustic signal enhancement at the tumor site 8 h after OA@Cho injection. The PAI results of isolated brain tissue were consistent with in vitro imaging, further confirming the specific accumulation of OA@Cho at the tumor site (Fig. 4f and g).

To further verify whether the targeting was due to ApoE adsorption, we achieved the reduction of cholesterol and ApoE levels in the blood of mice by continuous gavage of cholesterol-ApoE inhibitor glioma mouse model for two weeks (Fig. S4b–d), and then injected Free ICG, OA, and OA@Cho by tail vein to observe the fluorescence signals in the brain at different time points. The enrichment was assessed at different time points. It was obvious that no obvious fluorescent signals were observed in the brain of the OA group after the use of the inhibitor, while stronger fluorescent signals were still observed in the OA@Cho group (Fig. 4h and i), which on the other hand demonstrated that OA@Cho had a stronger adsorption capacity even when the ApoE content in vivo was lower, but was far inferior to the amount adsorbed when the ApoE content was normal. The isolated brain was also subsequently imaged and the same results were observed (Fig. 4j). The major organs of the mice were also imaged and quantified after deprogramming (Fig. S4e and f). These results indicate that OA@Cho is able to achieve effective BBB penetration by adsorbing ApoE and specifically accumulates at the glioma site, laying an important foundation for subsequent therapeutic studies.

### 3.5 Anti glioma effect in an orthotopic mouse model

**Fig. 5:**
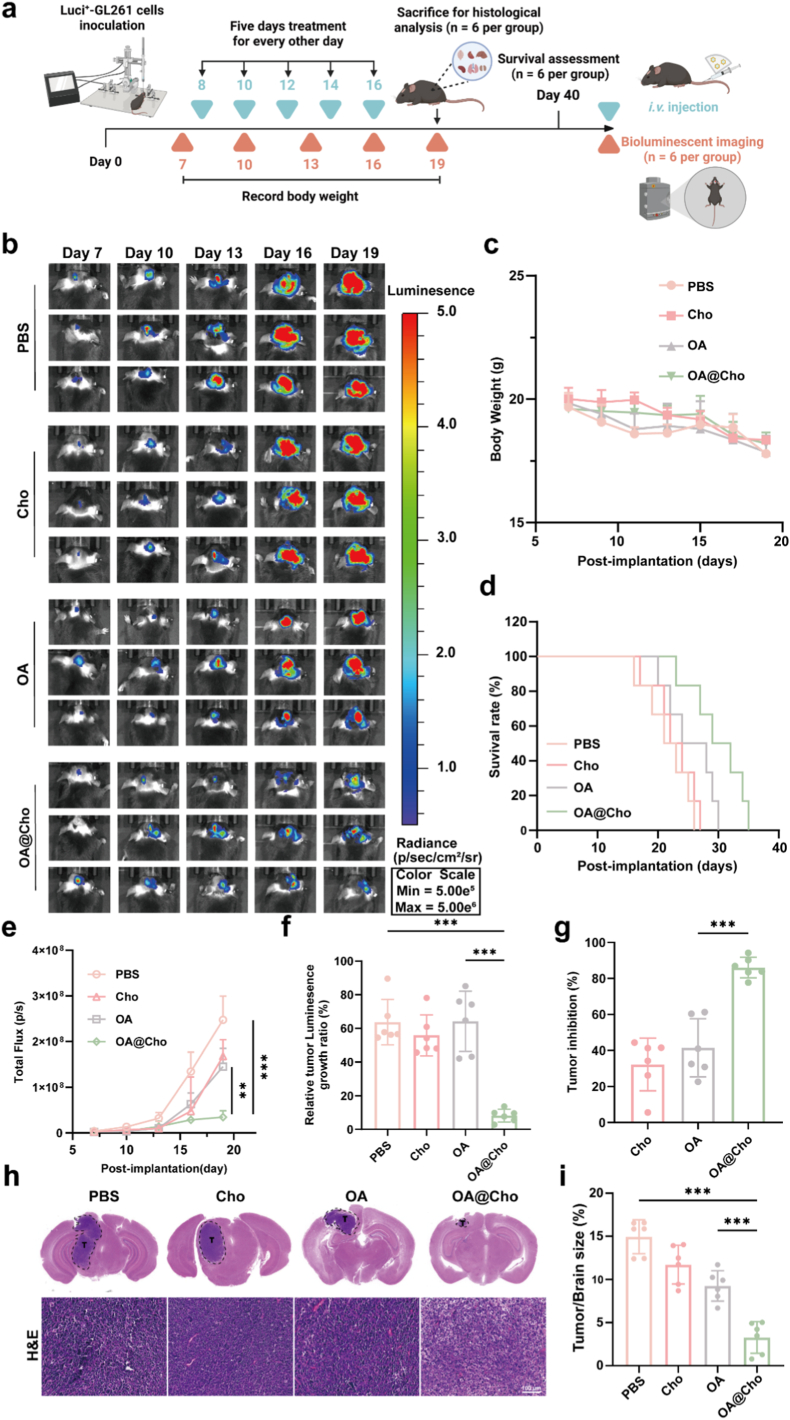
Antiglioma Efficacy of OA@Cho in an Orthotopic Glioma Model in vivo. a) Schematic illustration of the in vivo therapy timeline design. b) Biofluorescence imaging of Luc^+^-GL261 mice under different treatments. c) Weight monitoring of mice was performed every other day after treatment. d) KaplanMeier survival curves for different groups of mice. (n = 6, Kaplan‒Meier analysis, log-rank test). e) Quantification of bioluminescence intensity in groups of gliomas by the IVIS method. f) Relative tumor fluorescence growth ratios for different treatment regimens. g) Assessment of average tumor suppression. h,i) H&E-stained whole-sectional images of brain sections from each treatment group showing tumor growth and statistical analysis of tumor size (scale bar: 100 μm). Data are shown as the mean ± SD (n = 6). Two-way ANOVA were used to compare whether there are significant differences between data.

To evaluate the in vivo anti-tumor efficacy of OA@Cho, we established a Luc^+^-GL261 orthotopic glioma model. Tumor engraftment was confirmed by IVIS imaging 7 days post-stereotactic injection into the right striatum of female C57BL/6 mice. Mice were then randomized into four groups receiving intravenous injections of PBS, Cho, OA, or OA@Cho every 2 days. Tumor growth was dynamically monitored using in vivo bioluminescence imaging after intraperitoneal injection of D-luciferin on days 7, 10, 13, 16, and 19 (Fig. 5a). The results showed that the PBS and Cho-treated control mice showed a rapid growth trend, while the OA and OA@Cho-treated groups exhibited different degrees of tumor growth inhibition (Fig. 5b). Notably, the OA@Cho group exhibited the weakest bioluminescence signal on day 19 (Fig. 5e), and its TIR reached 86 %, which was significantly higher than that of other treatment groups (Fig. 5g). Quantitative analysis showed that tumor bioluminescence intensity increased by 70.38 %, 64.94 % and 58.86 % in the PBS, Cho and OA groups, respectively. while the OA@Cho group only increased by 16.18 % (Fig. 5f). This significant anti-tumor effect may stem from the specific targeting of gliomas by cholesterol-modified enhanced oncolytic adenovirus. Kaplan-Meier survival analysis further confirmed that OA@Cho treatment significantly prolonged the median survival of the tumor-bearing mice (Fig. 5d). During the treatment period, we assessed treatment-related toxicity by monitoring the change in body weight of mice. The results showed that the body weight of mice in all groups showed a trend of slow decrease, but there was no significant difference between groups (Fig. 5c), indicating that OA@Cho has good biosafety. The slow decrease in body weight may be related to the central nervous system dysfunction caused by the rapid progression of glioma. After the mice were euthanized on day 19, brain tissues were taken for pathological analysis. gross examination observation showed that the OA@Cho group had the smallest tumor volume, which was consistent with the biopsy results. H&E staining showed that the tumor tissues in the OA@Cho group presented obvious loose cellular structure and extensive tissue damage, while the tumor cells in the control group were morphologically intact (Fig. 5h and i). In summary, OA@Cho demonstrated significant antitumor effects in the Luc^+^-GL261 orthotopic glioma model, and its mechanism of action may involve various aspects such as enhanced tumor targeting, induction of tumor cell apoptosis, and inhibition of tumor cell proliferation.

### 3.6 Histological fluorescence imaging and OA@Cho induced immunogenic cell death

**Fig. 6:**
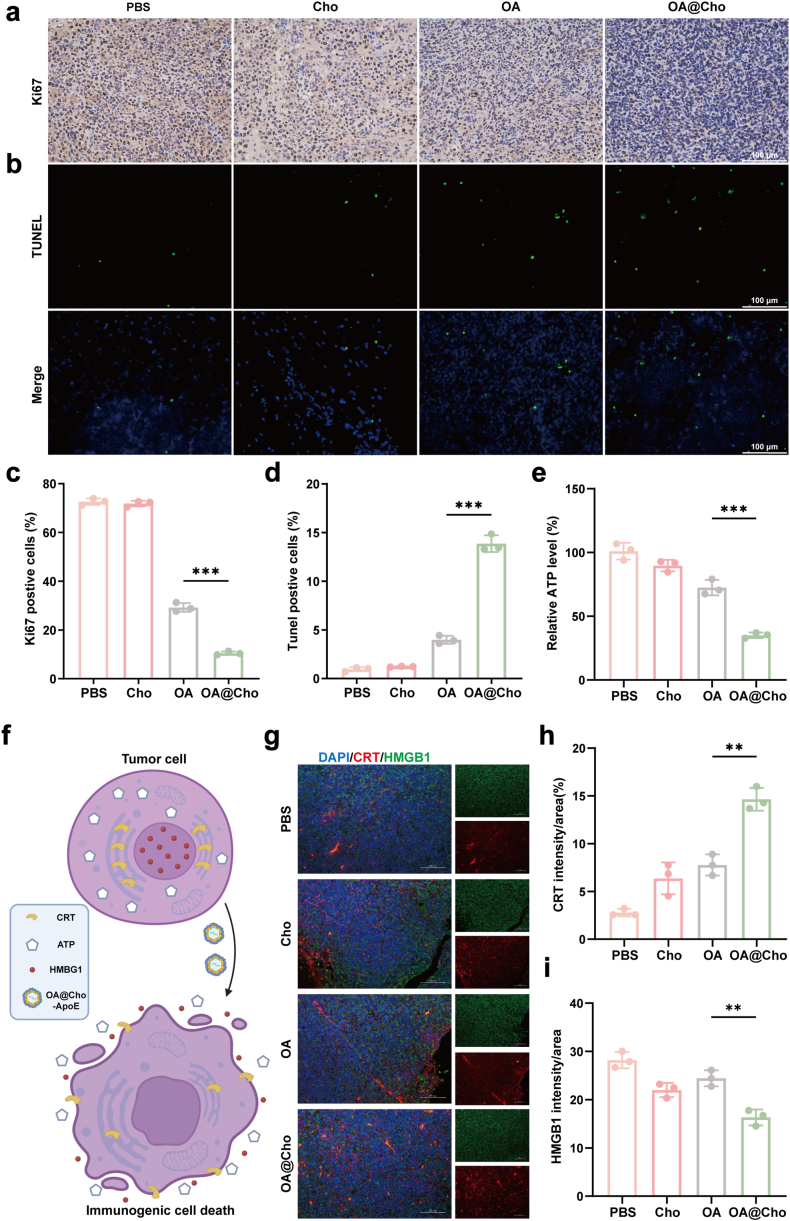
OA@Choinduced immunogenic cell death. a,b) Immunofluorescence staining of tumor tissue sections from orthotopic tumor-bearing mice by Ki67 and TUNEL. scale bar: 100 μm. c,d) Quantitative analysis of Ki67 and TUNEL positive cell levels. e) Detection of ATP release level. f) Schematic diagram illustrating OA@Cho-induced ICD in tumor cells. g) Representative immunofluorescence imaging of tumor sections stained for ICD markers. Scale bar: 100 μm. Data are presented as mean ± SD. (n = 3). Significant differences between indicated groups were calculated by one-way ANOVA and Tukey's multiple comparison test.

Immunohistochemical analysis showed that the rate of Ki67-positive cells in the OA@Cho group was significantly reduced to 10.46 % **(**Fig. 6a and c), while the rate of TUNEL-positive cells was significantly increased to 13.86 % (Fig. 6b and d), indicating that OA@Cho could effectively inhibit tumor proliferation and induce apoptosis. Beyond direct cytotoxicity, a key mechanism of oncolytic viruses like OA@Cho is the induction of ICD [44]. which we investigated next. Oncolytic viruses exert their therapeutic effects not only through direct tumor lysis but also by inducing ICD. This process releases tumor-associated antigens (TAAs) and damage-associated molecular patterns (DAMPs), which promote antigen-presenting cell (APC) maturation and amplify anti-tumor immunity (Fig. 6f) [45].We observed the release of high mobility group protein B1 (HMGB1), a DAMP normally localized to the nucleus where it functions in processes like proliferation and migration, into the extracellular space. Extracellular HMGB1 binds to receptors (e.g., TLR2/4, RAGE) on myeloid cells, including antigen-presenting cells, activating downstream signaling pathways that initiate an immune response. Immunofluorescence showed that HMGB1 fluorescence intensity was significantly reduced in tumor tissues of the OA@Cho-treated group, suggesting substantial release into the extracellular space. During ICD, CRT translocates from the endoplasmic reticulum to the cell surface, emitting an "eat-me" signal. OA@Cho-treated cells exhibited significantly enhanced CRT surface expression (Fig. 6g–i). During ICD, ATP is actively released into the extracellular space, acting as a chemoattractant for immune cells and promoting apoptotic cell clearance. Consistent with this release, OA@Cho treatment resulted in a significant decrease in intracellular ATP levels (Fig. 6e). These results confirm that OA@Cho is effective in inducing ICD and releasing DAMPs (HMGB1, CRT, ATP) and TAAs from tumor cells, a process that stimulates the maturation of immature dendritic cells (DCs) and enhances the antigen-presenting ability, thereby enhancing the efficacy of tumor immunotherapy.

### 3.7 Remodeling of the immune microenvironment in GBM mice

**Fig. 7:**
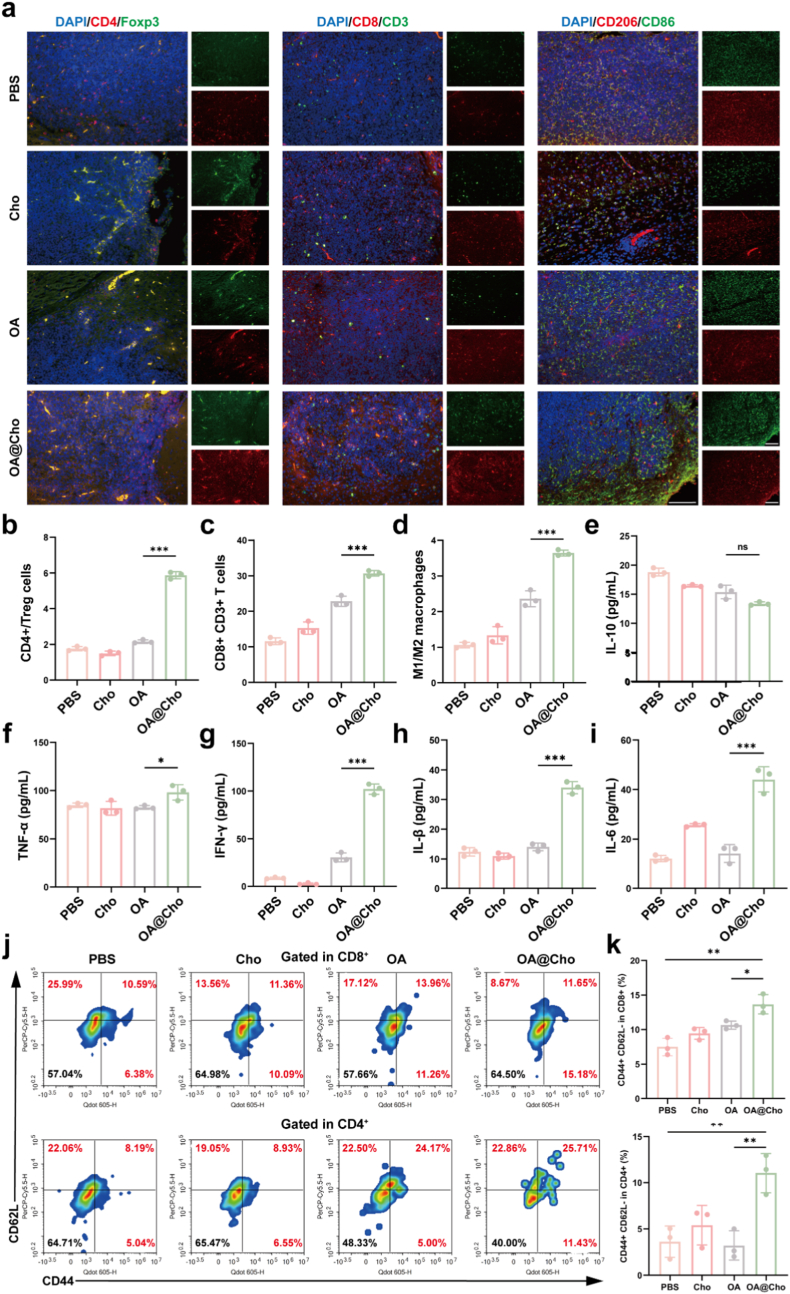
OA@Cho enhancement of anti-tumor immune response. a) IF staining images of CD4 (red) and Foxp3 (green). Blue: DAPI. Representative IF staining images of CD8 (red) and CD3 (green) in brain slices. Blue: DAPI. IF staining images of CD86 (red) and CD206 (green). Blue: DAPI. scale bar: 100 μm. b-d) Statistics on the ratio of CD4^+^ T cells to Treg cells, CD8^+^ T cells to CD3 cells, and M1/M2 cell. e-f) Determination of serum IL-10, TNF-α, IFN-γ, IL-1β and IL-6 levels in mice by ELISA assay. j,k) Flow cytometry analysis and quantification of naive T cells (CD44- CD62L+), central memory T cells (CD44⁺ CD62L⁺), and effector memory T cells (CD44⁺ CD62L⁻) in CD4⁺ and CD8⁺ T cells. Data are presented as mean ± SD. Two-way ANOVA were used to compare whether there are significant differences between data. (For interpretation of the references to colour in this figure legend, the reader is referred to the Web version of this article.)

We next investigated OA@Cho-mediated tumor microenvironment (TME) remodeling. OA@Cho converted the immunosuppressive TME to an immune-activated state by triggering anti-tumor immunity. IF staining of tumor tissues and results of revealed flow cytometry showed that the OA@Cho-treated group significantly increased the infiltration of CD3^+^ T cells and CD8^+^ T cells compared with the OA group, while decreasing the infiltration of immunosuppressive CD4^+^Foxp3+regulatory T cells (Tregs) (Fig. 7a–c, j, k; Fig. S5a–c). OA@Cho markedly altered macrophage polarization, increasing M1 macrophages while decreasing M2 macrophages (Fig. 7a–d). In addition to enhanced infiltration of effector T cells and reduced Tregs, these changes collectively reprogrammed TME into an immunologically activated state. In addition, we assessed the degree of systemic immune activation by ELISA for serum cytokine profiles. OA@Cho treatment significantly increased serum concentrations of pro-inflammatory cytokines—TNF-α, IFN-γ, IL-1β, and IL-6—compared to the OA group (Fig. 7f–i). The OA@Cho group exhibited significantly lower serum IL-10 levels (Fig. 7e). These cytokines are critical for DC maturation, cytotoxic T cell (CTL) initiation, and M1 macrophage differentiation [46], which together enhance overall anti-tumor immunity.

### 3.8 Mass cytometry characterizes the immune landscape of tumor in vivo by OA@Cho

**Fig. 8:**
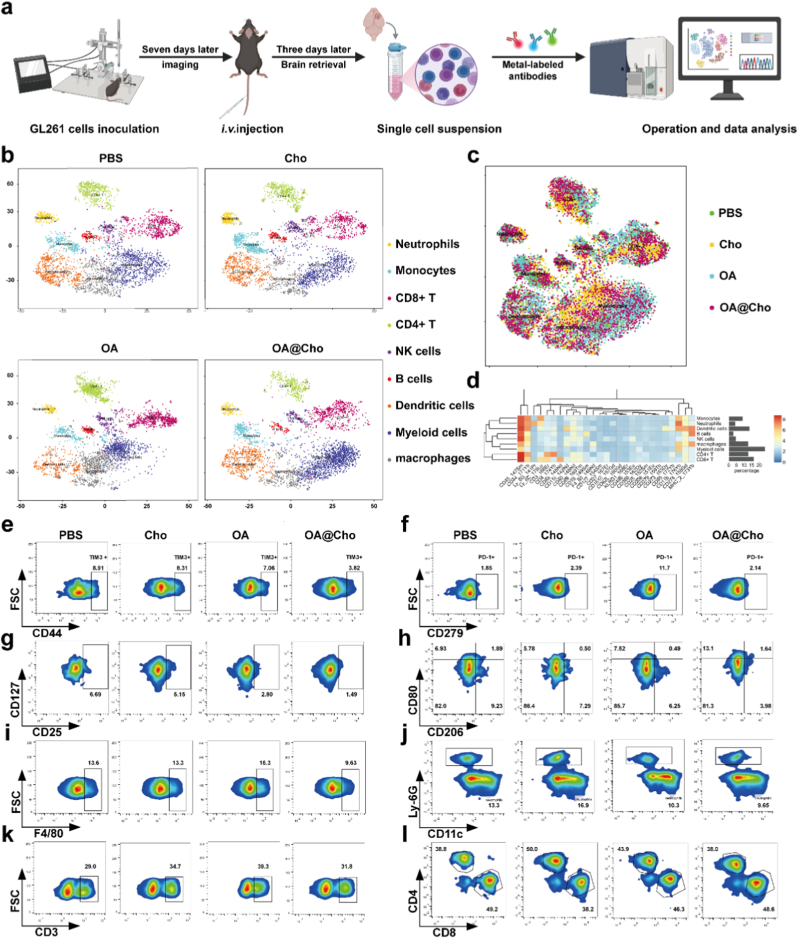
Mass cytometry of tumor-infiltrating immune cells. a) Schematic diagram of the CyTOF experimental procedure. b) The t-SNE profile of treatment with PBS, Cho, OA and OA@Cho(25,000 cells in each group). c) t-SNE plots of all samples (100,000 cells). d) Proportional statistics of the total number of sample clusters and heatmap of gene expression. e-l) Representative graphs of CyTOF analysis of immune cell infiltration after treatment of GBM mice (n = 6) with PBS, Cho, OA, and OA@Cho, as well as proportional analysis of CD44^+^ cells, CD279^+^ cells, CD25^+^CD127^+^ cells, M1/M2 ratios, macrophages, neutrophils, CD3^+^ cells, and CD8^+^CD4^+^ T cells. The numbers next to the drawn gates represent the percentage of cells.

To comprehensively assess the effect of OA@Cho on TME remodeling and anti-tumor immune response activation, we employed high-dimensional mass cytometry in GBM mouse model. Following tail vein injection of PBS, Cho, OA, or OA@Cho, brain immune cells were isolated from each treatment group (n = 6), pooled, and processed into single-cell suspensions. These suspensions were incubated with metal-conjugated antibodies and analyzed by CyTOF (Fig. 8a). Using the t-distributed stochastic neighbour embedding (t-SNE) dimensionality reduction model, we clustered 100,000 single cells into distinct immune cell subpopulations (Fig. 8c). The proportional distribution of these clusters across samples is visualized in Fig. 8b. After classifying immune cell types, samples were stratified by treatment group for correlation analysis of relevant genes. Finally, group-specific t-SNE profiles were generated (Fig. 8d). We further analyzed the specific proportions of immune cell subsets in OA@Cho-treated GBM (Fig. 8e–l). The ratio of CD44^+^TIM3^+^ to CD44^+^PD-1^+^ cells was significantly lower in the OA@Cho group compared to controls (Fig. 8e and f), suggesting cholesterol-modified viral vectors may possess enhanced immune evasion capabilities. Notably, while OA treatment alone elevated PD-1 expression, OA@Cho reversed this effect, further supporting its role in immune evasion. Additionally, the proportion of CD25^+^CD127^+^ cells was significantly reduced (Fig. 8g). Crucially, the M1/M2 macrophage ratio (CD80^+^/CD206^+^) increased dramatically by approximately 6-fold in the OA@Cho group (41.2 %) compared to the OA group (7.86 %) (Fig. 8h). Maturation of F4/80^+^ macrophages was also reduced following OA@Cho treatment (Fig. 8i). Myeloid-derived suppressor cells (MDSCs), known to accumulate and exert immunosuppression in tumor microenvironments, showed no significant changes between groups (Fig. 8j). The proportion of CD3^+^ T lymphocytes was significantly higher in both OA and OA@Cho groups compared to the PBS control, but lower in the OA@Cho group than in the OA group (Fig. 8k), suggesting OA@Cho treatment may moderately attenuate overall immune response intensity in vivo. Finally, statistical analysis of the CD8^+^/CD4^+^ T-cell ratio reaffirmed these findings (Fig. 8l).

These changes in the TME were closely related to our earlier observation of elevated levels of pro-inflammatory cytokines such as TNF-α, IFN-γ and IL-6 (Fig. 7f–i). These factors are known to drive DC maturation and M1-type macrophage polarization. Taken together, OA@Cho may effectively remodel the immunosuppressive "cold" GBM microenvironment into an immune-activated "hot" tumor by promoting DC maturation, inducing macrophage polarization towards M1-type, and depleting the "cold" GBM microenvironment. Promoting DC maturation, inducing macrophage polarization to M1-type, depleting immunosuppressive cells (e.g. MDSCs and tumor-associated macrophage TAMs), and expanding the infiltration of cytotoxic CD8^+^ T-cells and antigen-presenting DCs, thereby triggering an effective anti-tumor immune response.

### 3.9 In vivo safety evaluation

The systemic biocompatibility of nanoparticles is a key consideration for their biomedical applications. Ideal nano-delivery systems not only need to possess excellent pharmacological activity, but also need to ensure a high degree of biocompatibility [47]. In this study, the safety of intravenous administration of OA@Cho was comprehensively evaluated by systematic histopathological analysis and blood biochemical indexes. Histopathological analysis showed (Fig. S6a) that no significant histopathological changes were observed in vital organs such as heart, liver, spleen, lungs and kidneys in all groups of mice treated with PBS, OA, Cho and OA@Cho for 7 days, indicating that the experimental interventions did not trigger structural organ damage. The results of serum biochemical profiling further confirmed the systemic biocompatibility of the nanosystem. The key liver and kidney function indicators included serum albumin (ALB), alkaline phosphatase (ALP), alanine aminotransferase (ALT), aspartate aminotransferase (AST), total protein (TP), creatinine (CREA), lactate dehydrogenase (LDH), total bilirubin (TBIL), urea nitrogen (BUN), and uric acid (UA) (Fig. S6b–k), etc., and the concentrations of these indicators were within the physiological reference value range and did not differ statistically between groups. The concentrations of each index were within the normal murine physiological ranges and there was no statistical difference between the groups. Notably, hemolysis experiments showed that OA@Cho did not trigger significant hemolysis after 4 h of co-incubation with mouse erythrocytes, and the integrity of the erythrocyte membranes was well maintained, which further validated the systemic biocompatibility of the nanocomplexes from the perspective of hemocompatibility (Fig. S7a and b). In addition, we also examined the changes in Total Cholesterol (TC) levels in each group after 19 days of administration, and found that the serum TC levels in each treatment group did not differ and were within the normal range, and did not cause atherosclerosis and other hypercholesterolemic disorders (Fig. S7c). The above systematic evaluation demonstrated the safety of cholesterol-modified oncolytic adenovirus based on the delivery strategy of active adsorption of apolipoproteins in vivo in glioma orthotopic model mice.

Given that cholesterol in the circulation is mainly in the esterified form (more than 90 % of the total), the present study specifically focused on the quantitative analysis of lipoprotein isoforms before and after drug administration. The lipoprotein profile of total and free cholesterol showed that VLDL and LDL were maintained at baseline levels in both dosed and control groups, and the proportion of lipoprotein isoforms was not significantly altered between the groups (Fig. S8a–d). This finding has important biomedical implications: the cholesterol-modified nanocarriers did not interfere with the homeostasis of lipoprotein metabolism in vivo, which is consistent with their good compatibility profile in the blood circulation, and reaffirms the safety of the OA@Cho system for clinical application at the molecular level. Moreover, while this study focused on acute toxicity, the potential long-term metabolic effects of cholesterol modification warrant discussion. Our data show no acute dyslipidemia or tissue lipid accumulation (Figs. S6a, S7c, S8), likely due to the negligible pharmacological cholesterol dose used primarily for targeting. However, dedicated chronic toxicity studies remain essential for clinical translation and are a recognized future direction [32,47].

## 4 Discussion

Despite the promise of nanomedicine in oncology, the effective delivery of nanoparticles to GBM remains a paramount challenge. This is primarily due to the profound heterogeneity of the BBB in GBM [48]. As recently reviewed, this heterogeneity, characterized by the coexistence of leaky, compromised BBB regions and areas with intact, functional BBB, significantly undermines the reliability of the enhanced permeability and retention (EPR) effect, leading to unpredictable and inefficient nanoparticle delivery [48]. To overcome the inherent limitations of the passive EPR effect, active receptor-mediated transcytosis (RMT) has emerged as a promising strategy [49]. In line with this direction, our research indicates that the design of OA@Cho nanoparticles for adsorbing ApoE during in vivo circulation represents a promising strategy to overcome this limitation by enabling active LRP1/LDLR receptor-mediated transcytosis [50].

Our results demonstrate that cholesterol modification significantly enhances ApoE adsorption onto the viral surface (Fig. 1h–k). We propose that this selective enrichment is driven by altered surface chemistry: hydrophobic cholesterol "patches" preferentially recruit ApoE via its hydrophobic C-terminal lipid-binding domain, through strong hydrophobic interactions [31].This provides a more specific binding interface than the electrostatic-dominated non-specific adsorption on the naked virus. Thus, cholesterol modification actively skews the protein corona toward lipoproteins like ApoE, underpinning the selective enhancement. This biomimetic "surface modification-ApoE enrichment" strategy offers distinct advantages over conventional virus vector engineering for BBB penetration.

Specifically, compared to directly modifying vectors with targeting ligands for receptors such as transferrin receptor (TfR) [51]. our approach utilizes a more central nervous system-specific pathway through ApoE-LDLR/LRP1 interaction, potentially reducing off-target effects in peripheral tissues. Moreover, unlike strategies that rely on covalent coupling of targeted peptides (which are often static, costly, and may impair viral infectivity) [52], our cholesterol modification achieves a dynamic and efficient in vivo self-assembly process. This recruits natural full-length ApoE proteins that exhibit superior receptor binding affinity compared to short mimetic peptides [53], not only simplifying production but also establishing a universal platform expected to enhance the delivery of other therapeutic viral vectors.

When contextualized within the broader landscape of BBB-penetrating strategies, the profile of OA@Cho becomes clearer. Compared to physical methods (e.g., focused ultrasound) [54], OA@Cho is non-invasive and systemically applicable. Against biologicals like monoclonal antibodies, it offers a replicative and multimodal mechanism of action (oncolysis + immunotherapy). The pursuit of overcoming GBM's complex barriers is also driving other sophisticated nanoplatforms, such as hierarchically engineered nanocarriers designed to dynamically adapt to multiple biological barriers sequentially [55], bioorthogonal chemistry-activated "smart" systems for precise drug release [56], and sequential-targeting nanovaccines aimed at activating systemic immunity [57]. While these multi-step designs offer comprehensive solutions, our platform presents a simpler, yet highly effective alternative leveraging endogenous targeting mechanisms.

It is important to acknowledge several limitations of our strategy. Firstly, its efficacy is inherently dependent on endogenous ApoE levels, which may vary between individuals. Secondly, while our acute safety profile is promising, the long-term metabolic fate and potential immunogenicity of repeatedly administered cholesterol-modified viruses require further investigation. Lastly, the translational potential, particularly in the context of pre-existing immunity to adenoviruses, warrants careful evaluation in future studies.

Beyond improved delivery, the significance of OA@Cho lies in its ability to remodel the tumor immune microenvironment. The increased infiltration of cytotoxic T cells and shifted M1/M2 macrophage ratio, initiated by immunogenic cell death, underscore its potential as an effective immunotherapeutic agent. Our study, which utilizes a biomimetic nanoplatform to enhance the delivery and efficacy of an oncolytic virus, contributes to the expanding field of integrated nano-oncology [58]. This approach aligns with the broader trend of using nanotechnology to potentiate immunotherapy, a strategy applied not only to synthetic drugs and biologics but also to natural agents [59], sharing the common principle of engineering delivery systems to overcome biological barriers and modulate the immunosuppressive tumor microenvironment. This conceptual synergy highlights the universal potential of nanomaterial design in advancing cancer treatment paradigms.

## 5 Conclusion

In this study, a cholesterol surface-modified oncolytic adenovirus nanodelivery system (OA@Cho) was successfully constructed, and its physicochemical properties, targeting mechanism and anti-glioma efficacy were systematically elucidated. Through cholesterol modification, the protein corona thickness of OA@Cho was reduced from 15 nm to 5 nm, and the hydrated particle size was maintained below 190 nm, which met the physical requirements for blood-brain barrier penetration. The cholesterol modification significantly enhanced the specific interaction of the nanoparticles with ApoE, endowing them with the ability to target the LDLR family, and realizing a dual targeting effect on glioma cells and endothelial cells of blood-brain barrier. OA@Cho-ApoE significantly increased glioma cell uptake efficiency and effectively inhibited tumor cell energy metabolism and migration ability. In the blood-brain barrier model OA@Cho exhibited excellent trans-endothelial penetration performance and was able to penetrate deeply into 3D tumor spheroids. in vivo experiments further verified the therapeutic advantages of OA@Cho: through the ApoE-mediated active targeting mechanism, the accumulation of nanoparticles at the glioma site was enhanced compared with that of bare OA, which significantly prolonged the median survival period of the tumor-bearing mice, and the systematic safety assessment showed that the system did not cause any pathological damage to the vital organs, and the biochemical indexes of the blood and the metabolic homeostasis of lipoproteins were maintained at the normal level. This study has established an innovative three-in-one nanodelivery strategy of "surface charge modulation–apolipoprotein adsorption–receptor targeting". Within the TME, OA@Cho caused apoptosis and induced ICD by adsorbing ApoE in vivo, releasing TAAs and DAMPs and activating DCs and cytotoxic CD8^+^ T lymphocytes. Meanwhile, ApoE-enriched OA@Cho NPs reduced MDSCs and M2 macrophages while increasing M1 polarization, effectively converting immunosuppressed "cold" tumors into immune-active "hot" tumors, thereby enhancing anti-tumor immune response.

This provides a new technical pathway for oncolytic virus-targeted drug delivery therapy, as well as a theoretical basis for breaking through the blood-brain barrier, a key bottleneck for drug delivery in the central nervous system. Future research will focus on optimizing the density and spatial conformation of cholesterol modification to further improve the targeting precision and immune escape ability of the nanocomplexes, and to promote their translation to clinical applications.

## CRediT authorship contribution statement

**Aodi Niu:** Writing – review & editing, Writing – original draft, Visualization, Validation, Formal analysis, Data curation, Conceptualization. **Yuqing Lv:** Validation, Supervision. **Yuxuan Chen:** Validation. **Yuxuan Liu:** Validation, Investigation, Data curation. **Chengjian Luo:** Validation. **Mengqing Zheng:** Validation. **Yeyu Shen:** Validation. **Junjia He:** Validation. **Dongni Yao:** Validation. **Huanrong Lan:** Validation. **Hai Zou:** Validation, Supervision. **Tong Ge:** Validation. **Xiaozhou Mou:** Investigation, Funding acquisition, Formal analysis.

## Declaration of competing interest

The authors declare that they have no known competing financial interests or personal relationships that could have appeared to influence the work reported in this paper.

## Data Availability

Data will be made available on request.
