# Supplementary material for: Cholesterol surface-modified oncolytic adenovirus enriched with apolipoprotein E penetrates the blood-brain barrier to target glioblastoma immunotherapy
Source: Mater Today Bio. 2025 Sep 18;35:102319. doi: 10.1016/j.mtbio.2025.102319 (PMC12509134; doi:10.1016/j.mtbio.2025.102319)
Supplement: Multimedia component 1 [file mmc1.docx]

**Supplementary materials**

**Cholesterol Surface-Modified Oncolytic Adenovirus Enriched with Apolipoprotein E Penetrates the Blood-Brain Barrier to Target Glioblastoma Immunotherapy**

Aodi Niu^1,2^, Yuqing Lv^2^, Yuxuan Chen^2^, Yuxuan Liu^1^, Chengjian Luo^1^, Mengqing Zheng^1^, Yeyu Shen^2^, Junjia He^2^, Dongni Yao^2^, Huanrong Lan^5*^, Hai Zou^4*^, Tong Ge^3*^, Xiaozhou Mou^1,2*^

*^1^ School of Pharmacy, Hangzhou Normal University, Hangzhou 311121, China*

*^2^ Center for Rehabilitation Medicine, Clinical Research Institute, Zhejiang Key Laboratory of Tumor Molecular Diagnosis and Individualized Medicine, Zhejiang Provincial People's Hospital, Affiliated People's Hospital of Hangzhou Medical College, Hangzhou,310014, P. R. China*

*^3^ Emergency Department, Tiantai People’s Hospital of Zhejiang Province (Tiantai Branch of Zhejiang Provincial People’s Hospital), Hangzhou Medical College, Taizhou 317200, China*

*^4^Department of Critical Care, Shanghai Cancer Center, Fudan University, Shanghai 200032, PR China; Department of Oncology, Shanghai Medical College, Fudan University, Shanghai 200032, China*

*^5^Department of Surgical Oncology, Hangzhou Cancer Hospital, Hangzhou, Zhejiang 310002, China. No. 34, Yanguan Road, Hangzhou, 310002, Zhejiang Province, P. R. China*

E-mail: [getong@tmu.edu.cn](mailto:getong@tmu.edu.cn), [zouhai@fudan.edu.cn,](mailto:zouhai@fudan.edu.cn,) [lanhr2018@163.com,](mailto:xtsam@yahoo.com;) mouxz@zju.edu.cn


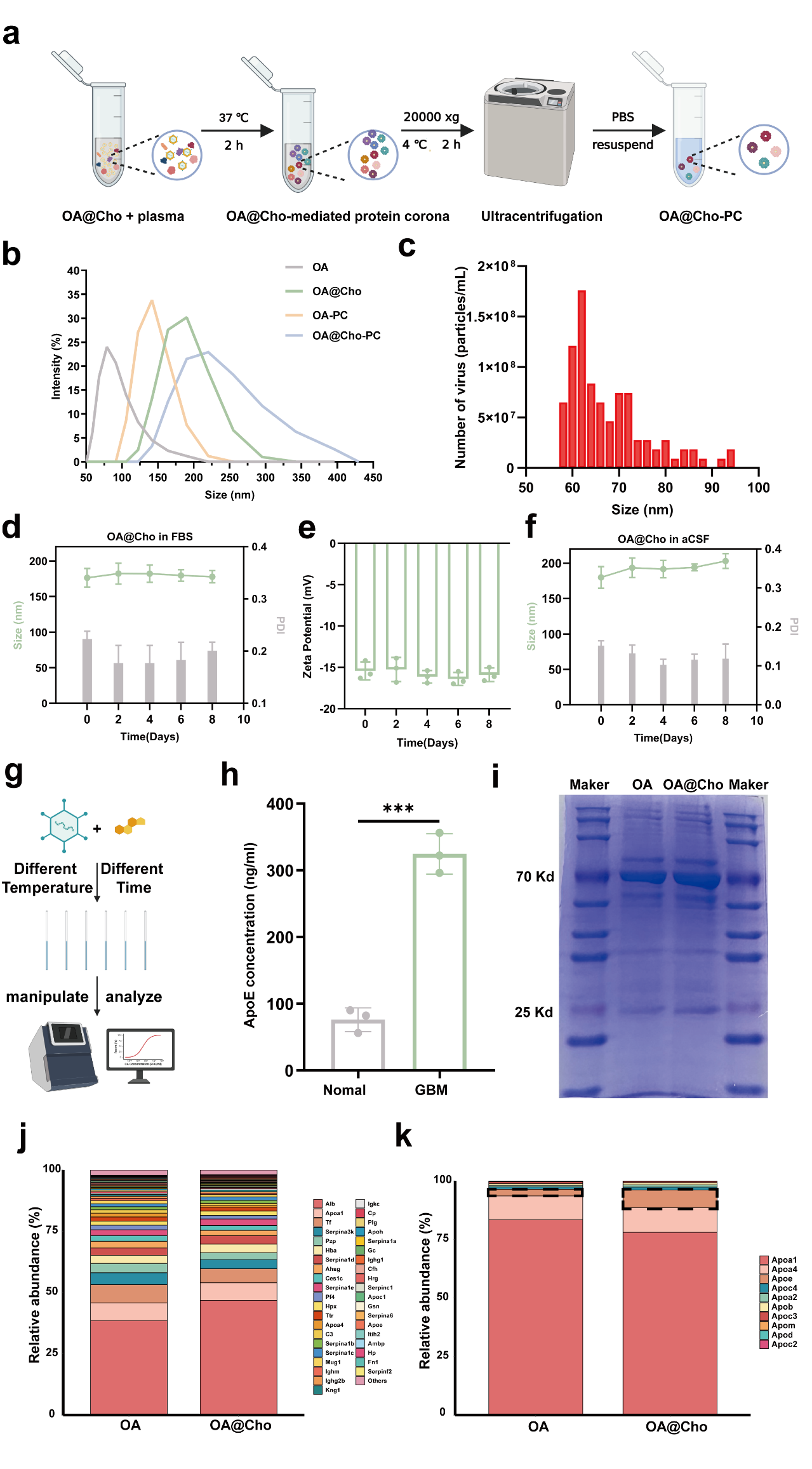


**Figure S1.** Particle size distribution curves. a) Schematic diagram of OA@Cho and protein corona preparation. b) Particle size distributions of OA, OA@Cho, OA-PC and OA@Cho-PC. c) NTA tests particle size distribution and number of viral particles in OA. d) Hydrodynamic diameter and PDI of OA@Cho nanoparticles incubated in DMEM supplemented with 10% FBS. at 37 °C over a period of 8 days. e) Zeta potentials of OA@Cho, at 37 °C over a period of 8 days. f) Hydrodynamic diameter and PDI of OA@Cho nanoparticles incubated in aCSF. at 37 °C over a period of 8 days. g) Schematic representation of the MST of the interaction between OA and cholesterol. h) Blood levels of ApoE in normal mice and glioma mice. i) Khomas-stained SDS-PAGE gel showing molecular weights of PC on OA and OA@Cho. j) The types and abundance of proteins adsorbed on OA and OA@Cho. k) The types and abundance of apolipoproteins on OA and OA@Cho.


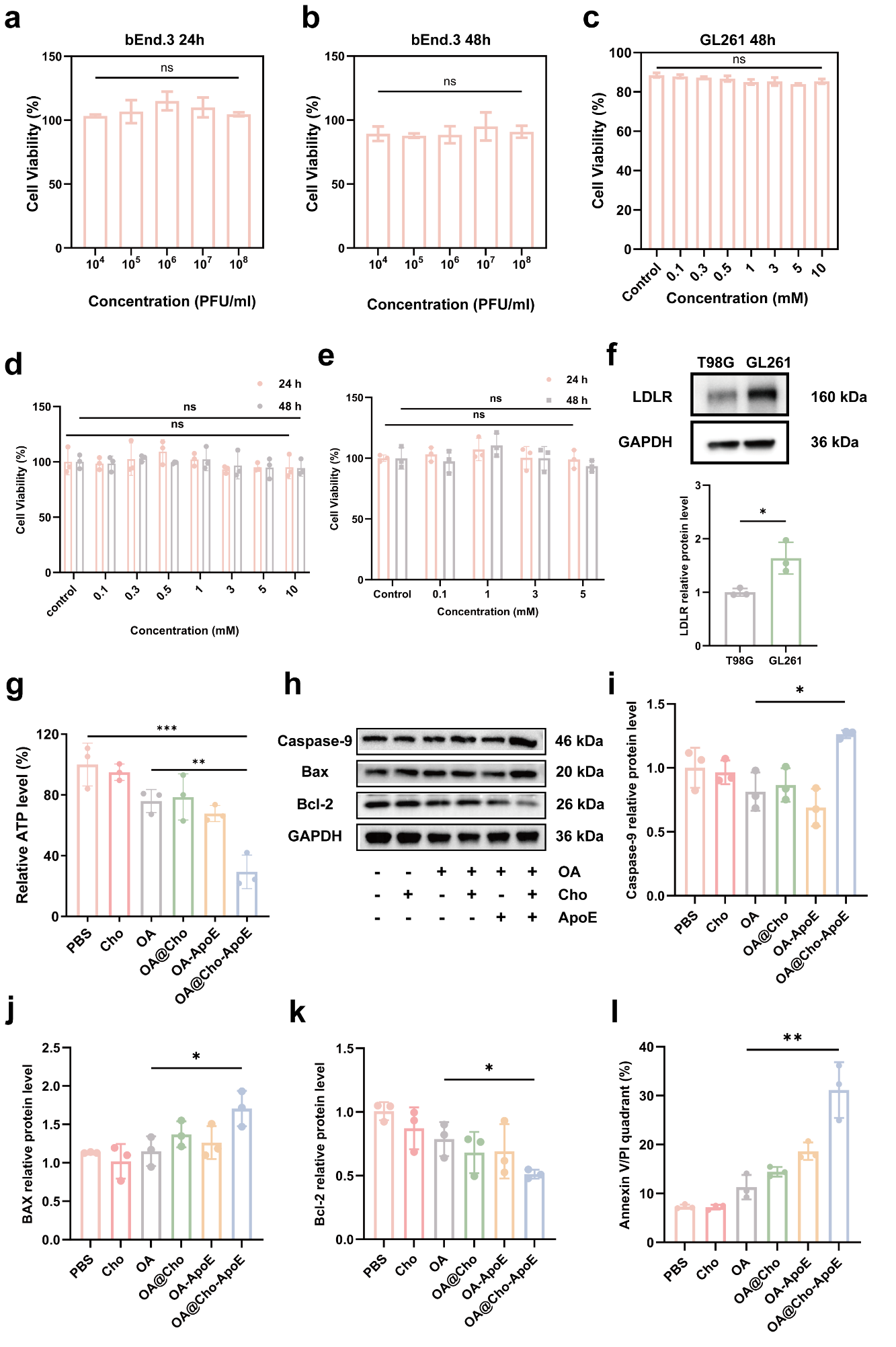


**Figure S2.** In vitro therapeutic performance. a, b) Effect of OA on cell viability of bEnd.3 cells at 24 h, 48 h. c) Effect of cholesterol on cell viability of GL261 cells at 48 h. d) Effect of cholesterol on cell viability of bEnd.3 cells at 24h, 48h. e) Effect of ApoE on cell viability of GL261 cells at 24h, 48h. f) Expression of LDLR protein in different glioma cell lines. g) After 24 hours of drug treatment, the intracellular ATP content changed. h-k) Expression levels of Caspase-9, Bax and Bcl-2 proteins after drug treatment. l) Quantification of apoptosis in GL261 cells induced by various treatments. Data are presented as mean SD and n = 3. T-test analysis was conducted to compare whether there were significant differences between the data.


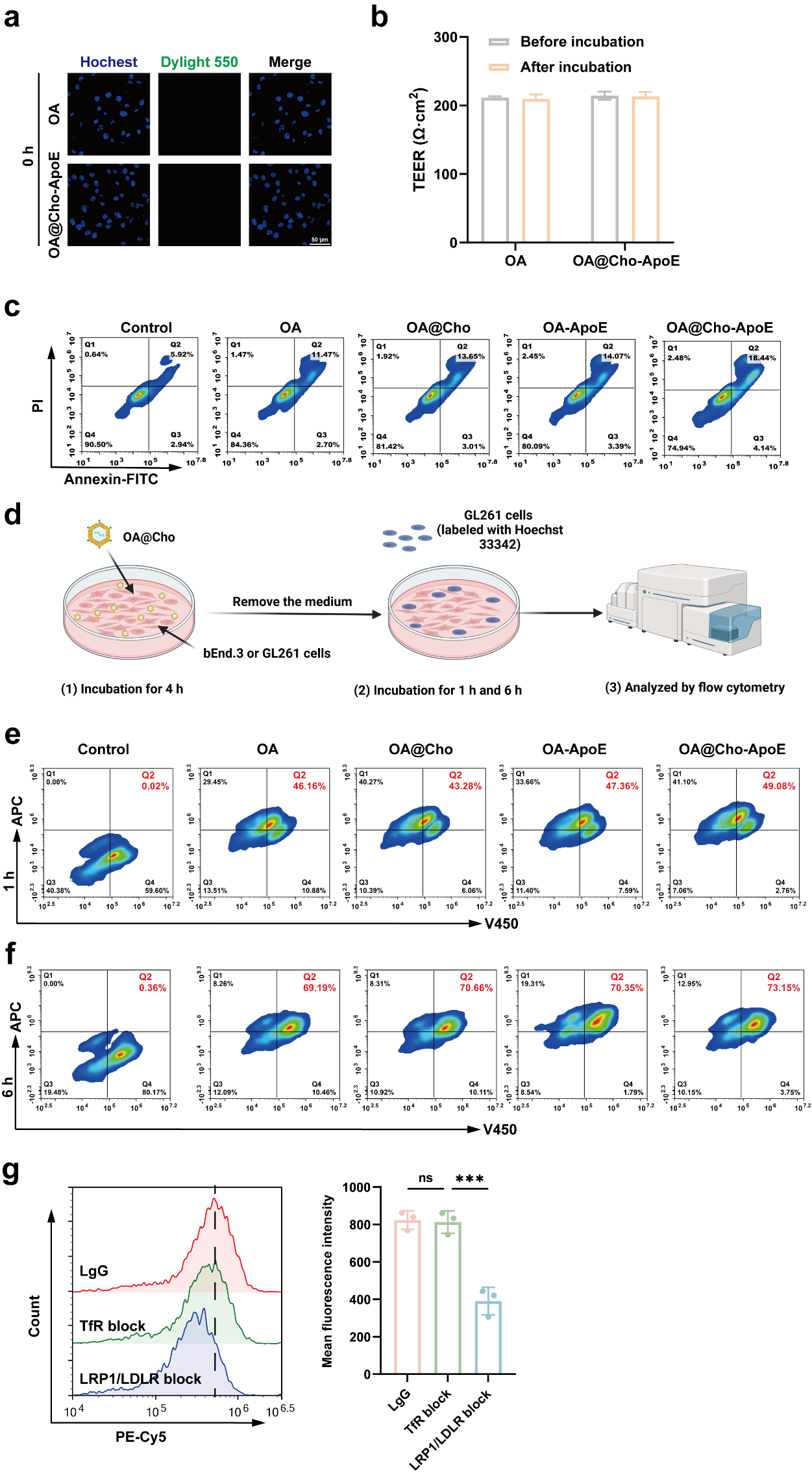


**Figure S3.** In vitro targeting of gliomas by OA@Cho adsorbed of ApoE. a) CLSM images of GL261 cells cultured with OA and OA@Cho-ApoE at 0 h. scale bar: 50 nm. b) Changes in TEER values before and after treatment in different groups (n = 3). c) Apoptosis of GL261 cells in the lower chamber after different treatment drugs was detected using flow cytometry. d) Schematic diagram of cellular transcytosis of OA@Cho analyzed by flow cytometry. e,f) Flow cytometry was used to determine the transcytosis efficiency of OA, OA@Cho, OA-ApoE, and OA@Cho-ApoE into GL261 cells after 1 h and 6 h of GL261 cells culture. Data are presented as mean SD and n = 3. g) Flow cytometry analysis of cellular uptake of OA@Cho-ApoE in GL261 cells with or without pre-treatment with an anti-LRP1/LDLR blocking antibody.


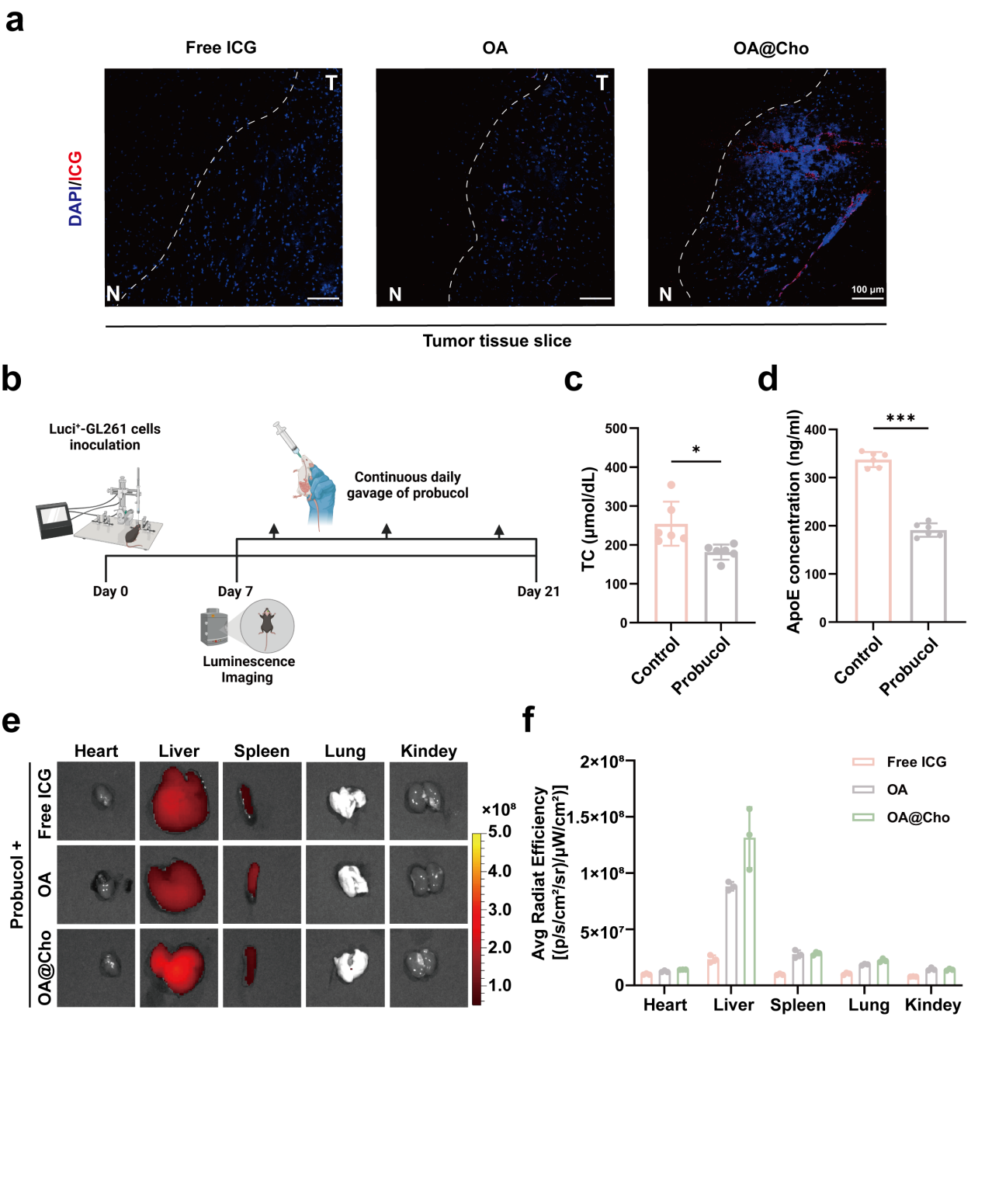


**Figure S4.** Targeting ability of OA@Cho under different conditions. a) CLSM observation of ICG fluorescence in localized mouse brain sections. scale bar: 100 µm. b) Construction of a glioma model with a schematic diagram of probucol gavage. c) Serum cholesterol levels in mice after 21 days. d) Serum concentration of ApoE in mice after 21 days. e,f) Fluorescence imaging of mouse organs after probucol gavage and its quantification. Data are presented as mean SD and n = 3.


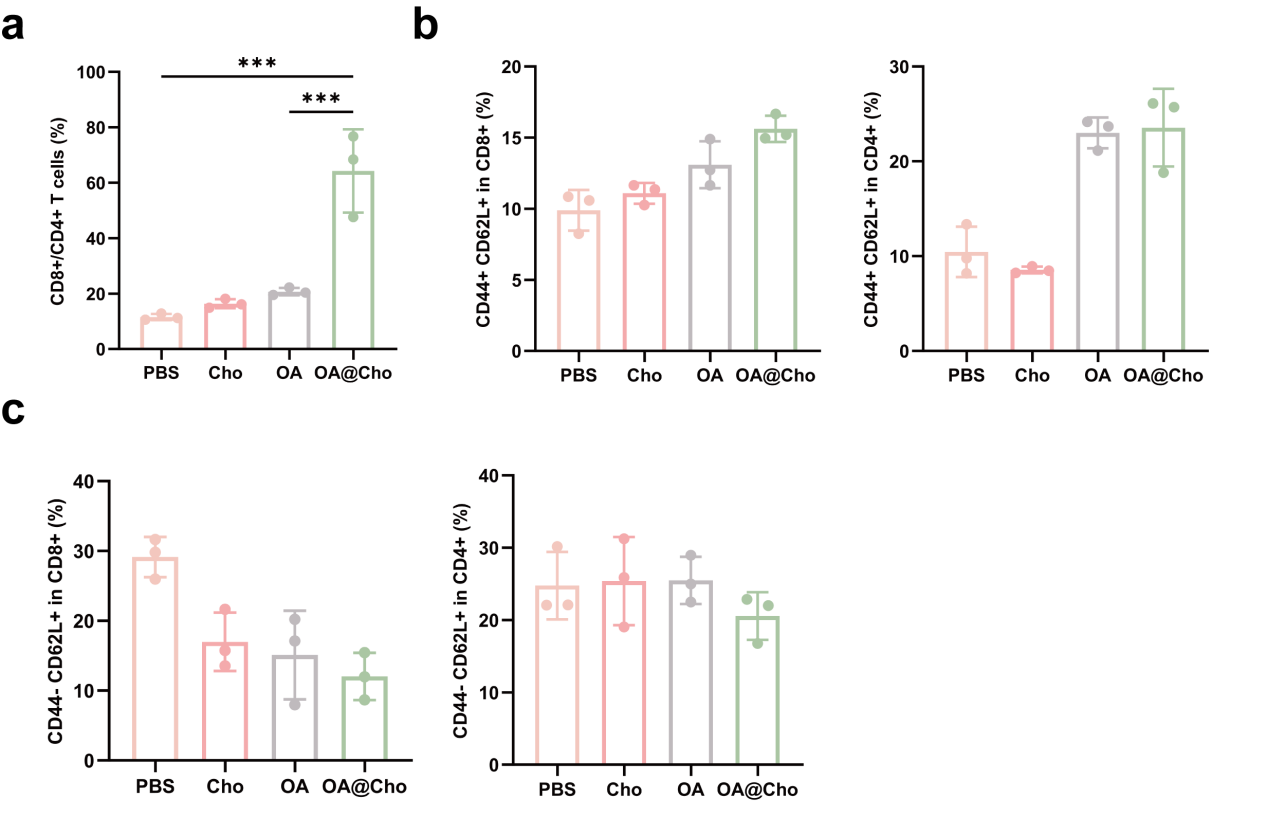


**Figure S5.** a) Flow cytometry analysis of CD8+/CD4+T cells. b) The proportion of CD44+CD62L - in CD8 and CD4 cells c) The proportion of CD44-CD62L+ in CD8 and CD4 cells. Data are presented as mean SD and n = 3.


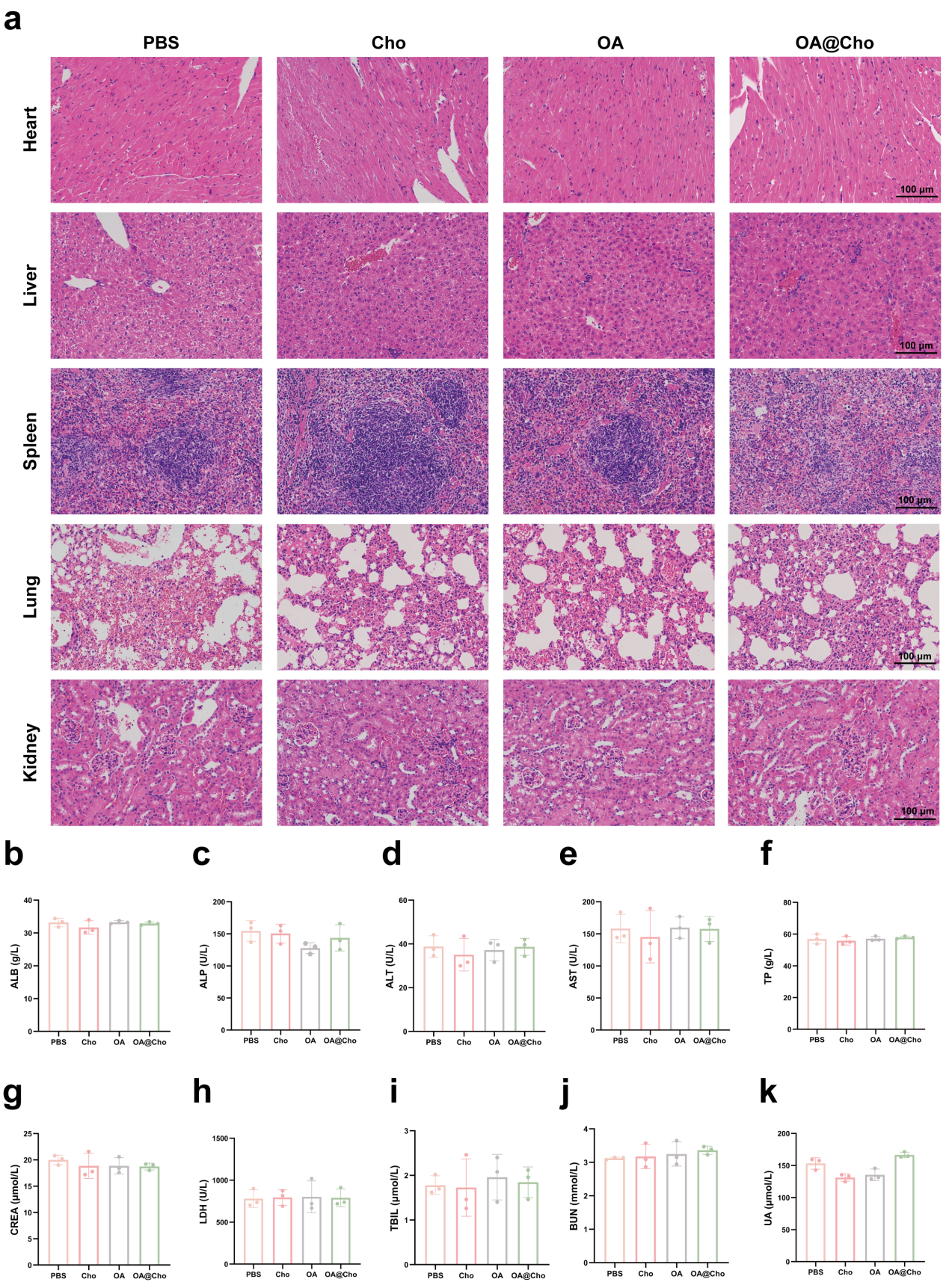


**Figure S6.** In vivo systemic biocompatibility evaluation. a) Histologic analysis of major organs (heart, liver, spleen, lungs, and kidneys) in glioma-bearing mice after treatment with PBS, Cho, OA, and OA@Cho. scale bar: 100 μm. b-k) Statistical analysis of ALB, ALP, ALT, ABT, TP, CREA, LDH, TBIL, BUN, UA. Data are presented as mean SD and n = 3.


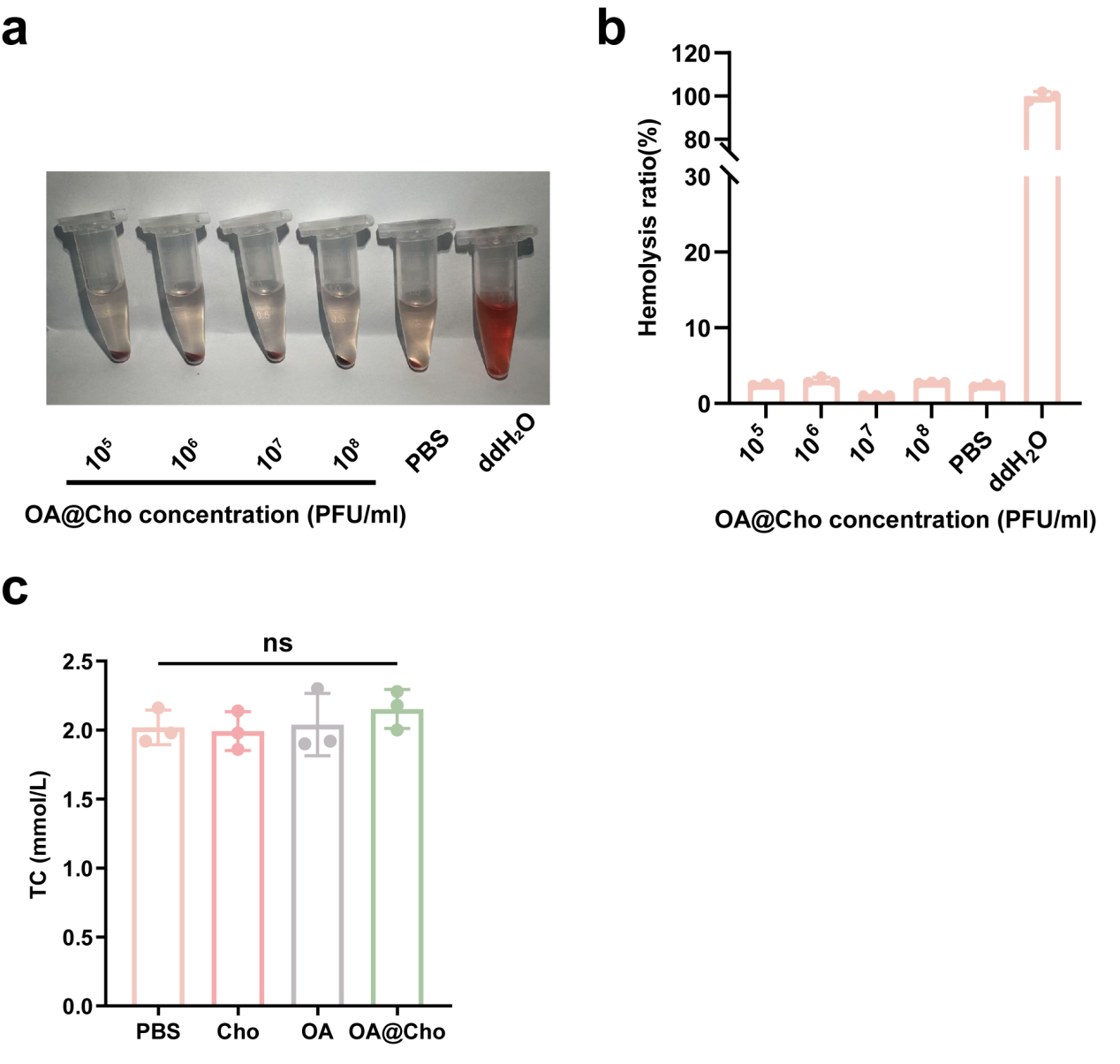


**Figure S7.** OA@Cho in vivo safety test. a,b) Haemolytic assay of OA@Cho. c) Levels of TC in serum of mice 19 days after administration of each treatment group. Data are presented as mean SD and n = 3.

**
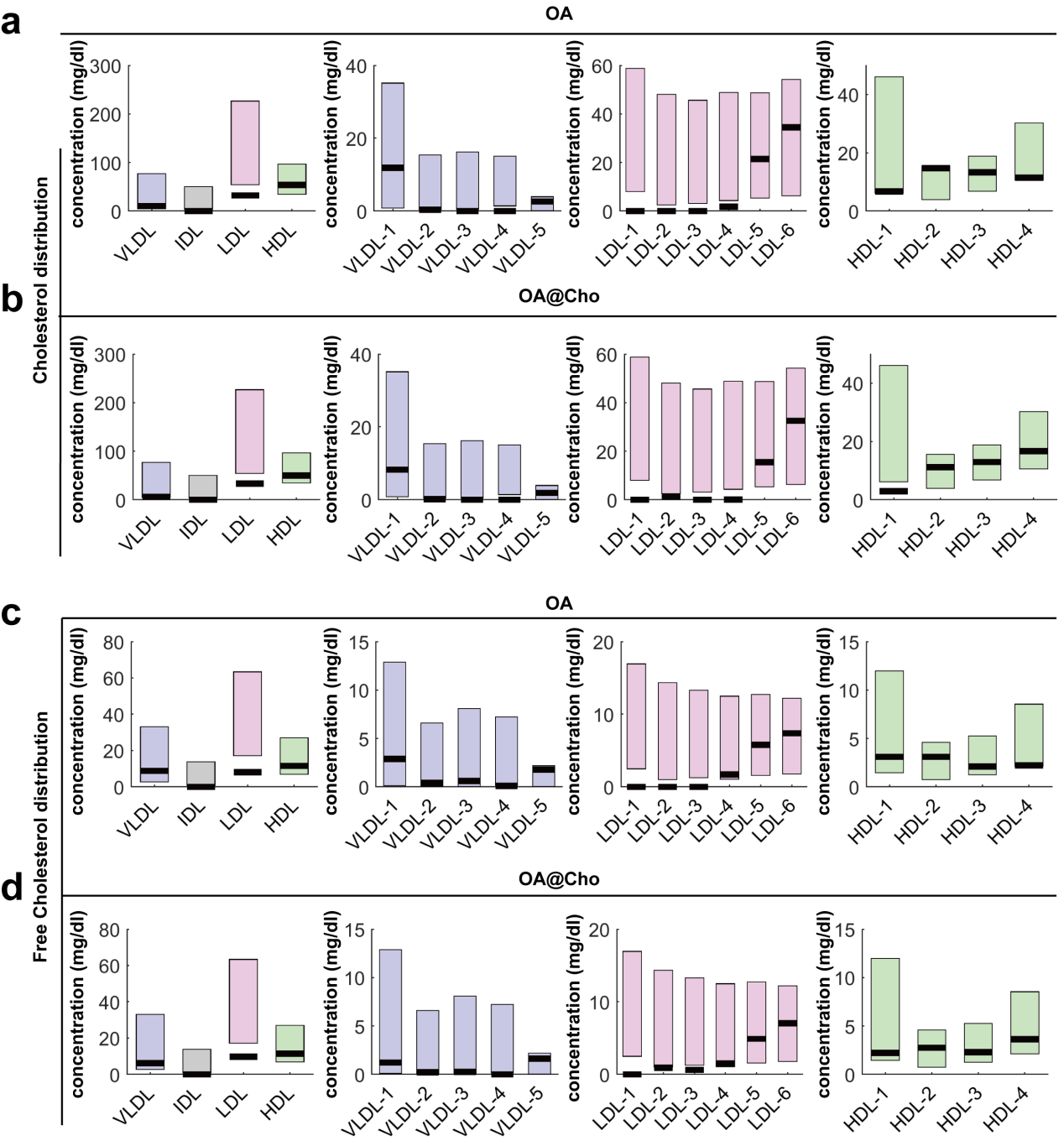
**

**Figure S8.** Analysis of the distribution of relevant lipid isoforms. a,b) Distribution of cholesterol-associated lipid isoforms before and after drug administration. c,d) Distribution of free cholesterol-associated lipid isoforms before and after drug administration.
